# Amelioration of diabetic nephropathy in mice by a single intravenous injection of human mesenchymal stromal cells at early and later disease stages is associated with restoration of autophagy

**DOI:** 10.1186/s13287-024-03647-x

**Published:** 2024-03-05

**Authors:** Jingjing He, Boxin Liu, Xiaofeng Du, Yan Wei, Desheng Kong, Baofeng Feng, Ruiyun Guo, Ernest Amponsah Asiamah, Matthew D. Griffin, Sean O. Hynes, Sanbing Shen, Yan Liu, Huixian Cui, Jun Ma, Timothy O’Brien

**Affiliations:** 1 Hebei Medical University-University of Galway Stem Cell Research Center, Hebei Medical University, Shijiazhuang, 050017 Hebei Province China; 2 Hebei Research Center for Stem Cell Medical Translational Engineering, Shijiazhuang, 050017 Hebei Province China; 3 Hebei Technology Innovation Center for Stem Cell and Regenerative Medicine, Shijiazhuang, 050017 Hebei Province China; 4 Hebei International Joint Research Center for Stem Cell and Regenerative Medicine, Shijiazhuang, 050017 Hebei Province China; 5 Human Anatomy Department, Hebei Medical University, Shijiazhuang, 050017 Hebei Province China; 6 Department of Forensic Sciences, College of Agriculture and Natural Sciences, University of Cape Coast, PMB UCC, Cape Coast, Ghana; 7 Regenerative Medicine Institute (REMEDI) at CÚRAM SFI Research Centre for Medical Devices, School of Medicine, University of Galway, Galway, Ireland; 8 Discipline of Pathology, School of Medicine, University of Galway, Galway, Ireland; 9 Department of Endocrinology, Hebei Medical University Third Affiliated Hospital, Shijiazhuang, 050051 Hebei China

**Keywords:** Mesenchymal stromal cells, Diabetic nephropathy, Cell therapy, Podocyte injury, Inflammation, Fibrosis, Autophagy

## Abstract

**Background and aims:**

Mesenchymal stromal cells (MSCs) a potentially effective disease-modulating therapy for diabetic nephropathy (DN) but their clinical translation has been hampered by incomplete understanding of the optimal timing of administration and in vivo mechanisms of action. This study aimed to elucidate the reno-protective potency and associated mechanisms of single intravenous injections of human umbilical cord-derived MSCs (hUC-MSCs) following shorter and longer durations of diabetes.

**Methods:**

A streptozotocin (STZ)-induced model of diabetes and DN was established in C57BL/6 mice. In groups of diabetic animals, human (h)UC-MSCs or vehicle were injected intravenously at 8 or 16 weeks after STZ along with vehicle-injected non-diabetic animals. Diabetes-related kidney abnormalities was analyzed 2 weeks later by urine and serum biochemical assays, histology, transmission electron microscopy and immunohistochemistry. Serum concentrations of pro-inflammatory and pro-fibrotic cytokines were quantified by ELISA. The expression of autophagy-related proteins within the renal cortices was investigated by immunoblotting. Bio-distribution of hUC-MSCs in kidney and other organs was evaluated in diabetic mice by injection of fluorescent-labelled cells.

**Results:**

Compared to non-diabetic controls, diabetic mice had increases in urine albumin creatinine ratio (uACR), mesangial matrix deposition, podocyte foot process effacement, glomerular basement membrane thickening and interstitial fibrosis as well as reduced podocyte numbers at both 10 and 18 weeks after STZ. Early (8 weeks) hUC-MSC injection was associated with reduced uACR and improvements in multiple glomerular and renal interstitial abnormalities as well as reduced serum IL-6, TNF-α, and TGF-β1 compared to vehicle-injected animals. Later (16 weeks) hUC-MSC injection also resulted in reduction of diabetes-associated renal abnormalities and serum TGF-β1 but not of serum IL-6 and TNF-α. At both time-points, the kidneys of vehicle-injected diabetic mice had higher ratio of p-mTOR to mTOR, increased abundance of p62, lower abundance of ULK1 and Atg12, and reduced ratio of LC3B to LC3A compared to non-diabetic animals, consistent with diabetes-associated suppression of autophagy. These changes were largely reversed in the kidneys of hUC-MSC-injected mice. In contrast, neither early nor later hUC-MSC injection had effects on blood glucose and body weight of diabetic animals. Small numbers of CM-Dil-labeled hUC-MSCs remained detectable in kidneys, lungs and liver of diabetic mice at 14 days after intravenous injection.

**Conclusions:**

Single intravenous injections of hUC-MSCs ameliorated glomerular abnormalities and interstitial fibrosis in a mouse model of STZ-induced diabetes without affecting hyperglycemia, whether administered at relatively short or longer duration of diabetes. At both time-points, the reno-protective effects of hUC-MSCs were associated with reduced circulating TGF-β1 and restoration of intra-renal autophagy.

**Graphical abstract:**

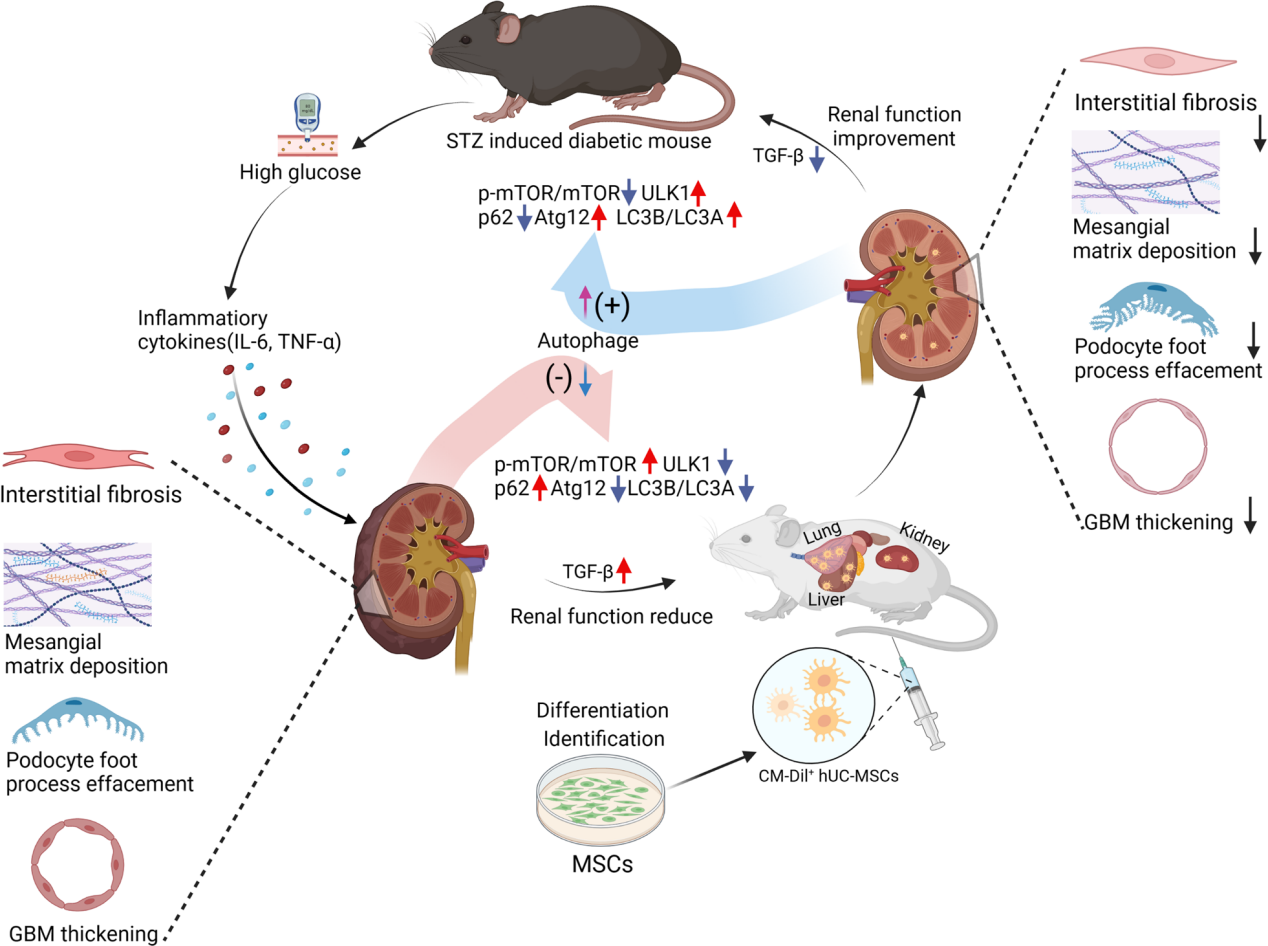

**Supplementary Information:**

The online version contains supplementary material available at 10.1186/s13287-024-03647-x.

## Introduction

The prevalence of diabetes mellitus (DM) has greatly increased in the past two decades, from 151 million people (4.6% of the global population) in 2000 to 537 million (10.5%) today. It is, furthermore, estimated that the number of people globally with DM in adulthood will increase to 643 million by 2030 (11.3%) and 783 million (12.2%) by 2045 [1]. Diabetic nephropathy (DN) is one of the most detrimental microvascular complications of DM, and occurs in both type 1 DM (T1DM) and type 2 DM (T2DM). Up to 40% of people with DM will eventually develop chronic kidney disease (CKD) as a result of DN and associated causes of kidney injury [2, 3].

Currently, the range of therapies with proven capacity to slow the progression of DN is limited to a small number of drug classes such as renin-angiotensin-system blockers, glucagon-like peptide-1 receptor agonists and sodium glucose transporter-2 inhibitors [4]. Although the introduction of each of these pharmacotherapies into routine clinical practice following large clinical trials has significantly improved the prognosis for patients with DM-associated CKD, their overall impact remains limited by the potential for adverse reactions, intolerance, drug interactions, variable responses and the cost implications of sequentially adding multiple drug classes [5, 6]. In addition, known pathophysiological mechanisms contributing to loss of kidney function in DM, such as chronic inflammation and fibrosis, are not directly targeted by currently available therapies [7]. There remains a significant need, therefore, to develop additional safe and effective strategies for the treatment of DN.

In this regard, it has been recognized for some time that local or systemic administration of stem/progenitor cells may have distinct beneficial effects in DN [8]. Mesenchymal stromal cells (MSCs) are a class of adult progenitor cells derived from mesoderm that have self-renewal capacity, multi-lineage differentiation potential and diverse pro-regenerative paracrine effects including extensively-characterized immunomodulatory properties [9–11]. A range of previous studies has reported potent beneficial effects of MSCs in experimental DN in mice, rats and other species. This pre-clinical literature has stimulated strong interest in clinical translation of MSC-based therapies for DN but, to date, the number of trials in this area has been low and no market-approved stem cell therapy has emerged. Among the challenges underlying this gap in translation are the need for better understanding of the disease stages at which MSC administration may be most effective linked to the identification of one or more consistent, reno-protective mechanisms of action of MSC-based therapies at each stage. In the current study, we aimed to compare and mechanistically characterize the beneficial effects of a single intravenous dose of human umbilical cord-derived MSCs (hUC-MSCs) on kidney abnormalities at an early and a later stage of the mouse streptozocin (STZ)-induced model of DM. While MSCs may be isolated from a number of sources, we selected hUC-MSCs for the study based on their potential clinical advantages of ease of collection, low immunogenicity, high capacity for culture-expansion and distinctive paracrine properties [12, 13].

Autophagy is a cellular self-protection mechanism that helps maintain the homeostasis of podocytes, glomeruli, mesangial cells, and tubules at normal blood glucose levels. Disordered autophagy in renal cells in diabetes mellitus has been shown to be a component of the pathophysiology of DN [14–16]. For that reason, we sought to investigate whether the administration of MSCs would restore diabetes induced disordered autophagy and thus might be an important therapeutic target in DN.

## Methods

*Experimental animals* Male C57BL/6 mice with the weight 23–26 g (6–7 weeks old) were purchased from Beijing HFK Bio-Technology Co., Ltd. and were housed in the specific pathogen free (SPF) animal breeding room of Hebei Medical University. All animals were housed at constant temperature (20 ± 2 °C) and humidity (45–55%), with a 12:12 h light: dark cycle and with standard diet and water ad libitum. This study adheres to the ARRIVE guidelines for the reporting of animal experiments.

*Ethics statement* All animal procedures were performed in accordance with the guidelines for the Care and Use of Laboratory Animals and the Animal Welfare Act in China and approved by the Committee of Ethics on Experimentation of Hebei Medical University (Permit Number: 2021035). The experiments were conducted according to the National Institutes of Health (NIH) Guidelines for the Care and Use of Laboratory Animals.

*Establishment of animal models* After 1 week acclimatization, DM was induced in male mice by intraperitoneal (i.p.) injection of 80 mg/kg STZ (Cayman Chemicals, catalog no. 18883664) in 0.1 M citrate buffer, at pH 4.5 following 6 h fasting for 5 consecutive days, as described previously [17, 18]. Non-diabetic mice received an equal volume of citrate buffer according to the same schedule. For all animals, blood glucose was measured following 6 h of fasting via tail vein with an Accu-Chek Go glucometer (Roche Diagnostic, Mannheim, Germany). Mice with fasting blood glucose (FBG) level > 16.7 mmol/L at 72 h and 7 days post-STZ injection were confirmed as having DM and were used in the study. FBG and weight were monitored at the same time of day (14:00) starting at 6 or 11 weeks after initiation of STZ/citrate buffer injections. The 24-h urine was collected via metabolic cage from 7 or 15 weeks. General distress scoring (GDS) was performed weekly by two researchers to monitor the health status of the mice [19]. All efforts were made to minimize the animals’ suffering, with pre-defined humane endpoints (Additional file 1: Table S1).

*Culture and characterization of UC-MSCs* Human UC-MSCs were purchased from Qilu Cell Therapy Technology (Shandong, China) and were cultured according to the supplier’s recommended protocol. Cryopreserved hUC-MSCs were transferred to complete medium and cultured in a humidified 37 °C, 5% CO_2_ incubator. The medium was changed after 2–3 days, and then exchanged every 3 days thereafter. When the cells had reached 80–90% confluence rate, the culture medium was discarded, the flasks were washed three times with PBS, and the cells were detached with MSC digestion solution (Jing-Meng, Beijing, China). The cells were passaged at a rate of 1:3, and characterization was carried out at passage 4.

The expected immunophenotype was confirmed by flow cytometry of a suspension of passage 4 cells for the surface marker proteins CD73, CD44, CD29, CD105, CD90, CD45, and HLA-DR using the BD Biosciences Human MSC Analysis Kit (catalog no. 562245, BD Biosciences, Franklin Lakes, NJ, USA) according to the manufacturer-recommended protocol. Mouse isotype control antibodies were used to define positive and negative staining. Flow cytometry was performed on a BD FACS Calibur (BD Biosciences) and the resulting data files were analyzed using FlowJo Software (Treestar, Ashland, OR).

For differentiation assays, hUC-MSCs were cultured in 6-well plates in adipogenic differentiation (Cyagen Biosciences, Guangzhou, China, catalog no. HUXUC-90031) or osteogenic differentiation media (Cyagen Biosciences, Guangzhou, China, catalog no. HUXUC-90021). For chondrogenic differentiation, hUC-MSCs were seeded at passage 4 in 15 ml sterile centrifuge tubes in chondrogenic differentiation medium (Cyagen Biosciences, Guangzhou, China, catalog no. HUXUC-90041). The induction of differentiation was performed using a standard protocol from Cyagen Biosciences Inc. Oil red O staining was used to confirm adipogenesis, Alizarin red staining was used to verify osteogenesis, and Alcian blue staining was used to examine chondrogenesis. Human UC-MSCs cultured under baseline conditions at the same passage number served as negative controls in all three differentiation assays.

*Labelling of UC-MSC for *in vivo* bio-distribution study* For an in vivo biodistribution study, hUC-MSCs were labeled with chloromethyl-1,10-dioctadecyl-3,3,30-tetramethylindo-carbocyanine perchloride (CM-Dil, Molecular Probes, Eugene, OR, USA, catalog no. C7000) as previously described [20]. Briefly, 5 × 10^5^ passage 4 hUC-MSCs were suspended in 1 mL of 1 mg/mL CM-Dil working solution at 37 °C in 5% CO_2_ for 5 min, and then for an additional 15 min at 4 °C. The cells were shielded from light and intermittently mixed during the labelling reaction. After the above steps, hUC-MSCs were washed twice with phosphate-buffered saline (PBS) then re-suspended in fresh medium directly.

**Fig. 1:**
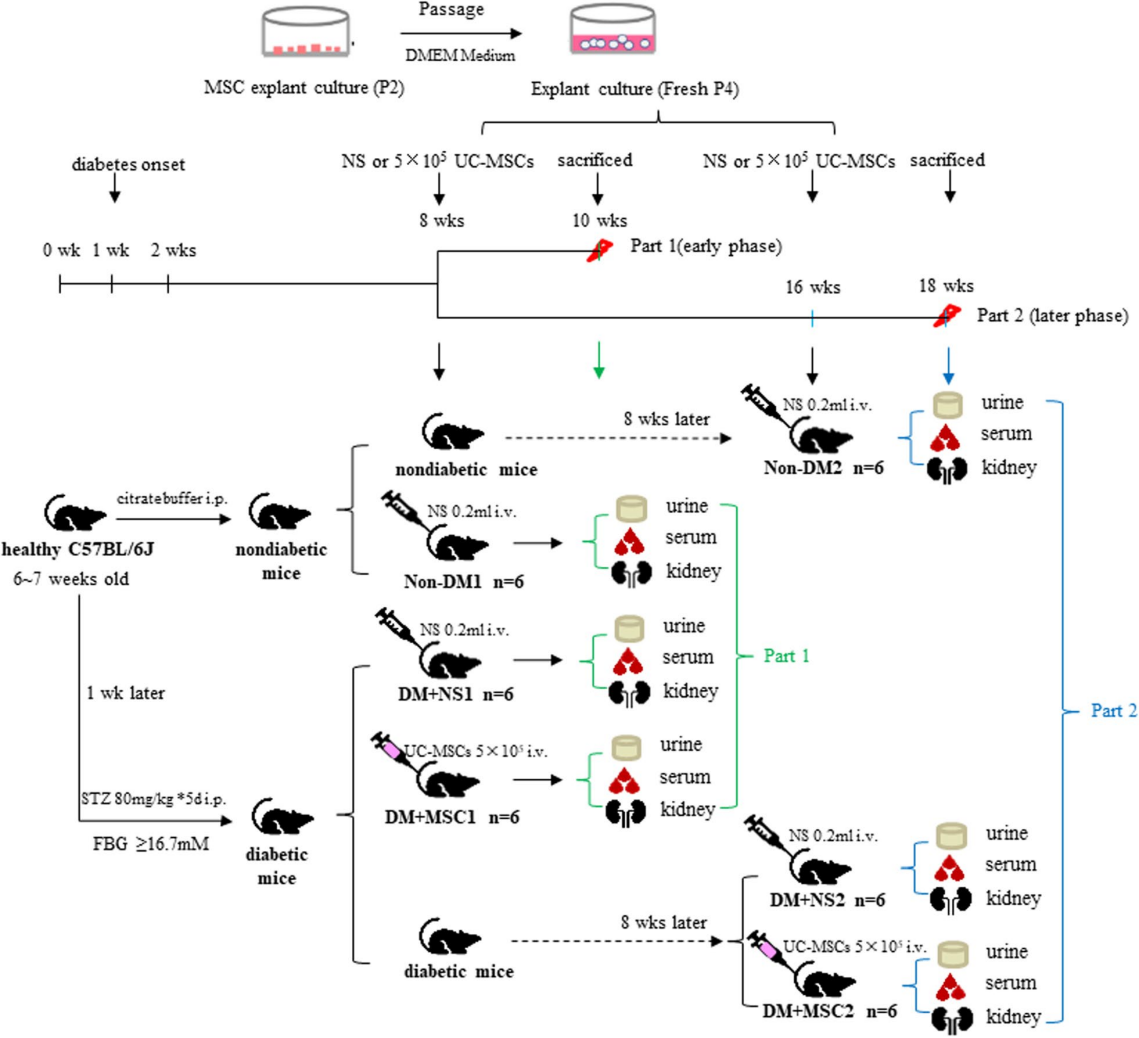
Designs of In vivo Experiments Diagram summarizing the designs of two experiments for investigating the effects of hUC-MSCs on kidney disease in the C57BL/6 mouse model of streptozotocin (STZ)-induced diabetes mellitus. As shown, mice were injected intra-peritoneally (i.p.) with 80 mg/kg STZ (diabetic/DM) or citrate buffer (nondiabetic/Non-DM) for 5 consecutive days. One week later, DM was confirmed by fasting blood glucose (FBG) > 16.7 mM. Eight weeks and 16 weeks after the injection of STZ/citrate buffer, groups of 6 DM and non-DM mice received 0.2 mL intravenous injections of normal saline (NS) or 5 × 10^5^ human umbilical cord-derived mesenchymal stromal cells (hUC-MSCs) as illustrated. Two weeks later (10 weeks or 18 weeks following initiation of STZ/citrate buffer injections), mice were euthanized and urine, serum and kidneys were collected for analyses

In vivo* experiments involving UC-MSC administration* The in vivo studies of the effects of hUC-MSC administration in diabetic mice consisted of two independent experiments, each consisting of 3 groups of 6 animals (see Fig. 1 for illustrations of the experimental protocols).

*Experiment 1 (early time-point hUC-MSC administration)*: For this experiment, 18 mice that had initiated the STZ-induced diabetes (DM) or control (non-DM) protocol 8 weeks previously were assigned to the following 3 groups:

Group I (Non-DM1): Non-DM mice injected i.v (via tail vein) with 0.2 mL normal saline (NS).

Group II (DM + NS1): DM mice injected i.v with 0.2 mL NS.

Group III (DM+MSC1): DM mice injected i.v. with 0.2 mL containing 5 × 10^5^ hUC-MSCs.

All mice were monitored closely for a further 2 weeks, following which they were euthanized and samples of biological fluids and tissues were collected for analyses. In subsequent data presentations, results for these groups are referred to as “10 weeks”, reflecting the total duration of DM prior to sample analysis.

*Experiment 2 (later time-point hUC-MSC administration)* For this experiment, 18 mice that had initiated the STZ-induced diabetes (DM) or control (non-DM) protocol 16 weeks previously were assigned to the following 3 groups:

Group I (Non-DM2): Non-DM mice injected i.v (via tail vein) with 0.2 ml normal saline (NS).

Group II (DM + NS2): DM mice injected i.v with 0.2 mL NS.

Group III (DM+MSC2): DM mice injected i.v. with 0. 2 mL containing 5 × 10^5^ hUC-MSCs.

For both experiments, DM mice were assigned randomly to NS and MSC groups. For all mice treated with hUC-MSCs, passage 4 cells were used. The selected cell dose was determined through preliminary experiments (data not shown) and immunosuppressive agents were not administered.

All mice were monitored closely for a further 2 weeks, following which samples of urine and blood were collected and animals were humanely euthanized for tissue collection. In subsequent data presentations, results for these groups are referred to as “18 weeks”, reflecting the total duration of DM prior to sample analysis.

*Biological distribution of UC-MSCs* In a separate experiment, a total of 8 mice were injected i.v., via tail vein with 0.5 × 10^6^ CM-Dil-labelled hUC-MSCs in 0.2 mL of NS. At 24 h, 48 h, 7 days and 14 days later, 2 mice were euthanized as described above and the lungs, liver, and kidneys were removed by dissection, frozen in Tissue-Tek OCT (Sakura FineTek, Tokyo, Japan) and sectioned at 8 μm thickness using a cryostat microtome (Leica CM3050 S, Nussloch, Germany). Sections were incubated at room temperature for 30 min in 10% blocking serum (Jackson Laboratories, CA, USA) and then stained with 40,6-diamidino-2-phenylindole, dihydrochloride (DAPI, Vector Laboratories, Southfield, MI, USA). Stained kidney sections were analyzed by immunofluorescent microscopy (DP74, Olympus, Japan) to detect and capture images of CM-Dil^+^/DAPI^+^ hUC-MSCs at different time-point following i.v. injection.

*Urine, serum and tissue collection* Two weeks after NS/hUC-MSC injections, mice were placed individually into metabolic cages (Suzhou Fengshi Laboratory Animal Equipment Co., Ltd, Suzhou, China) for 24 h with free access to food and water for urine volume quantification and collection of urine samples for biochemical analyses. The 24-h urine samples were centrifuged and used to quantify urine albumin and urine creatinine concentrations using a Hitachi 7600 automated chemistry analyzer (Hitachi limited, Japan) based on which urine albumin-creatinine ratios (uACR) were calculated. A total urine albumin excretion of 30 mg/24 h was considered abnormal and evidence of DN.

After urine collections were completed, mice were anesthetized by inhalation of 2 to 3% isoflurane following a 6 h fast with free access to water and blood samples were collected by abdominal aortic puncture. Once a blood clot had formed, blood was centrifuged at 1200×*g* for 15 min at 4 °C to generate serum, which was aliquoted to 200 μL in individual sterile tubes and stored at − 80 °C until use for biochemical and cytokine assays. The mice were then euthanized by decapitation and the kidneys were removed by abdominal dissection. The left kidneys were weighed, then 1 mm^3^ pieces of renal cortex were quickly cut on ice and fixed in 2.5% glutaraldehyde in 0.1 M phosphate buffer (pH 7.4) for transmission electron microscopy (TEM). The remaining tissue from the left kidneys was flash frozen in liquid nitrogen and stored at − 80 °C. The right kidneys were bisected longitudinally and fixed overnight at 4 °C in 4% paraformaldehyde (Solarbio Science & Technology, Beijing, China) then paraffin embedded for subsequent histological studies. The kidney index (KI) was calculated as the ratio of the left kidney weight (KW) to the body weight.

*Renal histopathology* Three to four micrometer thick sections of paraffin-embedded kidney tissue were prepared using a microtome for each kidney (n = 6 kidneys per group). Individual sections were stained with hematoxylin and eosin (H&E), periodic acid-Schiff (PAS), and Masson’s trichrome staining using standard protocols (Additional file 2: Supplemental methods). Images were collected at 200× magnification and the stained sections were analyzed in blinded fashion using a light microscope. Quantitative image analysis was performed using Image-Pro Plus 6.0 software (Image-pro Plus, Media Cybernetics, Inc., USA).

*TEM* Ultrastructural glomerular features were analyzed in glutaraldehyde-fixed cortical tissue samples using a Hitachi HT7700 transmission electron microscope (Hitachi High-Technologies Corporation, Japan). Podocyte foot processes and glomerular basement membrane (GBM) were observed at a 25,000 magnification. GBM thickness was defined as the distance perpendicular to the GBM between the endothelial and podocyte plasma membranes, and was measured by point-to-point methods with Image-Pro Plus 6.0 software. Three different segments of glomerular basement membrane per mouse from six mice were measured with a total of 18 measurements in each group [21].

*Immunohistochemistry* For immunohistochemical staining, 4-μm-thick sections of paraformaldehyde -fixed, paraffin-embedded kidney tissue were dewaxed in water, then antigen retrieval was performed in citric acid buffer (pH 6.0) by the high-pressure method. Next, the ZSGB-bio kit (ZSGB-BIO, Beijing, China, catalog no. SP-9001) was used for immunostaining according to the manufacturer’s instructions. First, sections were blocked with 3% H_2_O_2_ for 10 min with shielding from light then blocked with donkey serum for 30 min at room temperature. Subsequently, sections were incubated overnight at 4 °C with rabbit anti-Wilm’s tumor 1 monoclonal antibody (WT1; 1:100, Abcam, Cambridge, UK, catalog no. ab89901), followed by incubation with HRP-labeled goat anti-rabbit IgG secondary antibody (Jackson ImmunoResearch, West Grove, PA, USA, 1:500) for 10 min in the dark. After washing with PBS and counterstaining with 3, 3’-diamnobenzidine (DAB), the sections were passed through concentration gradients of alcohol and xylene to dehydrate. The stained sections were analyzed and images were captured using a DP74 light microscope (Olympus). WT1-positive cells on the outer aspect of the GBM were considered to be glomerular visceral epithelial cells (podocytes) and were counted. WT1-negative cells or cells in the inner aspect of the GBM were not counted. Podocytes were counted using Image-Pro Plus 6.0 software in blinded fashion for between 15 and 20 randomly-selected glomerular profiles (at the hilar level) in each longitudinal kidney section in high-power magnification (400× magnification) [22].

*Enzyme-linked immunosorbent assay (ELISA)* Serum concentrations of interleukin (IL)-6 (abclonal, Wuhan, China, catalog no. RK00027) and tumor necrosis factor alpha (TNF-α) (abclonal, Wuhan, China, catalog no. RK00027) and transforming growth factor beta 1 (TGF-β1) were measured using ELISA kits (abclonal, Wuhan, China, catalog nos. RK00008, RK00027 and RK00057 respectively) according to the manufacturers’ instructions. At the completion of the ELISA protocols, the optical density of each well at 450 nm wavelength was quantified with a microplate reader and the final serum concentrations were calculated according to a standard curve.

*Western blot* For each kidney, approximately 10 mg of frozen tissue were lysed in 100 μL of ice-cold RIPA lysis buffer (Beyotime, China) containing 1mM phenylmethylsulfonyl fluoride (Beyotime, China). The concentration of protein was determined by bicinchoninic acid (BCA assay). For protein detection, 30 μg of each protein lysate were loaded in 10–12% SDS-PAGE gels which were subjected to electrophoresis and transferred onto polyvinylidene difluoride (PVDF) membranes (Millipore, USA). After blocking with 5% skim milk for 2 h, the membranes were respectively incubated overnight at 4°C with antibodies against LC3A/B (1:1,000, Abcam, UK, ab48394), Atg12 (1:1,000, Cell Signaling Technology, USA, #4180), SQSTM1/p62 (1:1000, Cell Signaling Technology, USA, #8025), ULK1 (1:1000, Proteintech, USA, 20986-1-AP), mTOR (1:1000, Cell Signaling Technology, USA, #2983) and phospho (p)-mTOR (Ser2448) (1:1000, Cell Signaling Technology, USA, #2971).Next, the PVDF membranes were incubated with corresponding secondary antibodies (1:10,000, servicebio, China) for 2 h at room temperature. Specific protein bands were developed using enhanced chemiluminescence kits and detected with a ChemiDoc™ XRS + system (Bio-Rad, USA). After one target protein was developed, the mild stripping buffer was used to strip Western blots according to the stripping for reprobing protocol (Abcam, UK, ab282569) and the β-actin antibody (1:1,000, servicebio, China) was incubated in this PVDF membrane to detect relative protein expression. Image J software was used for quantitative analysis of two independent bands, and the densitometry of the target bands was expressed as a mean of each band, normalized to housekeeping protein, β-actin (n = 6 independent samples).

*Statistical analysis* All data are presented as mean ± SEM. Statistical analysis was performed using SPSS 18.0 (SPSS, Chicago, IL, USA) and GraphPad Prism 9.3 (La Jolla, CA, USA). Shapiro–Wilk test was firstly used for normal distribution assay, and all data were following normal distribution. For multiple group comparisons, one-way analysis of variance (ANOVA) with Tukey's multiple comparison test were performed. *P* value < 0.05 was considered statistically significant.

## Results

**Fig. 2:**
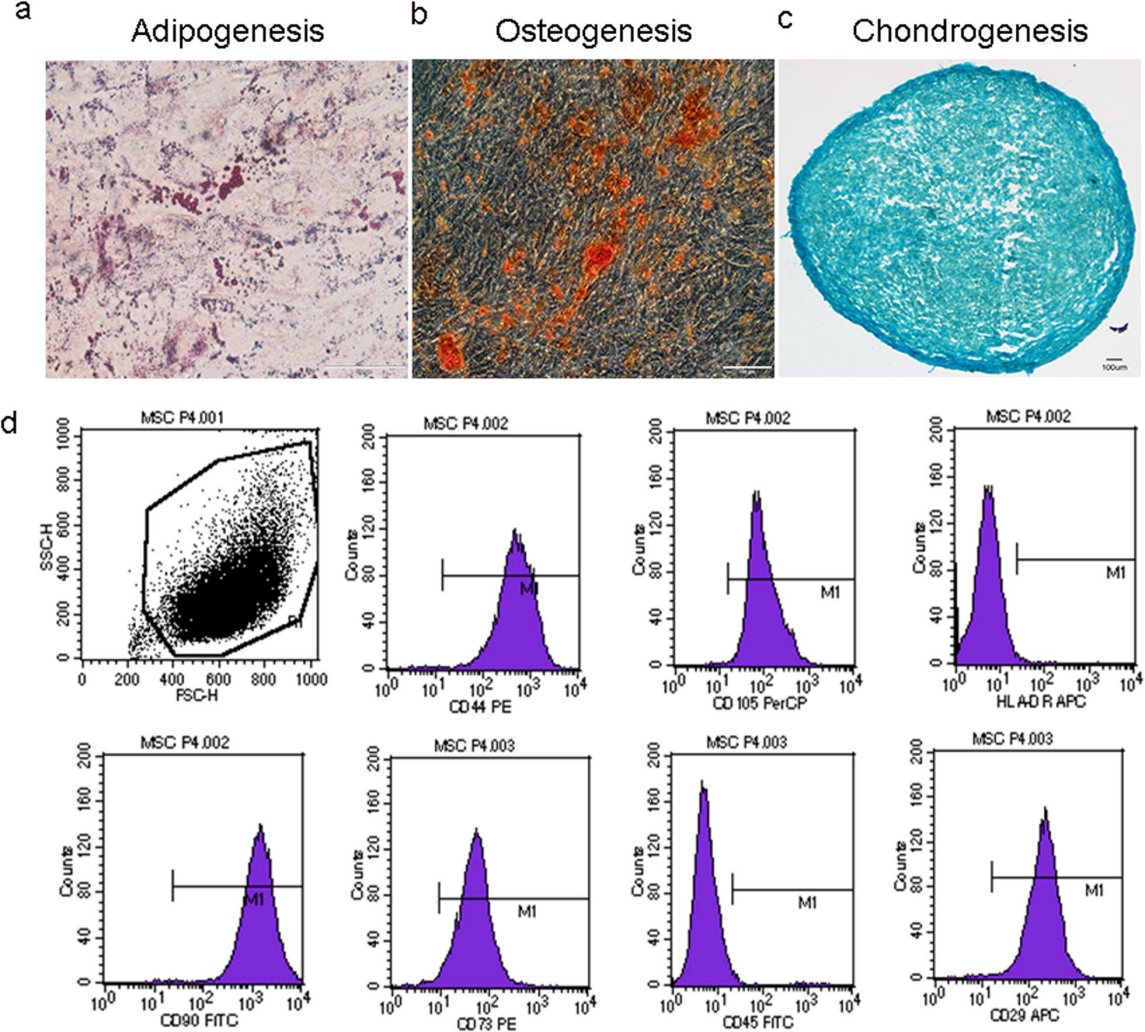
Differentiation and Surface Marker Profile of hUC-MSCs: **a**–**c** Representative photomicrographs demonstrating **a** Oil red O staining of lipid droplets in hUC-MSCs under adipogenic culture conditions, **b** Alizarin red staining of hUC-MSCs under osteogenic culture conditions and **c** Alcian blue staining of hUC-MSC pellets cultured under chondrogenic conditions. Scale bar, 100 μm. **d** Representative flow cytometry dot plot demonstrating hUC-MSC scatter characteristics and histograms demonstrating relative fluorescence following surface staining with monoclonal antibodies against: CD90, CD73, CD45, CD29, CD44, CD105 and HLA-DR. Differentiation and surface marker expression experiments were repeated in triplicate

**Fig. 3:**
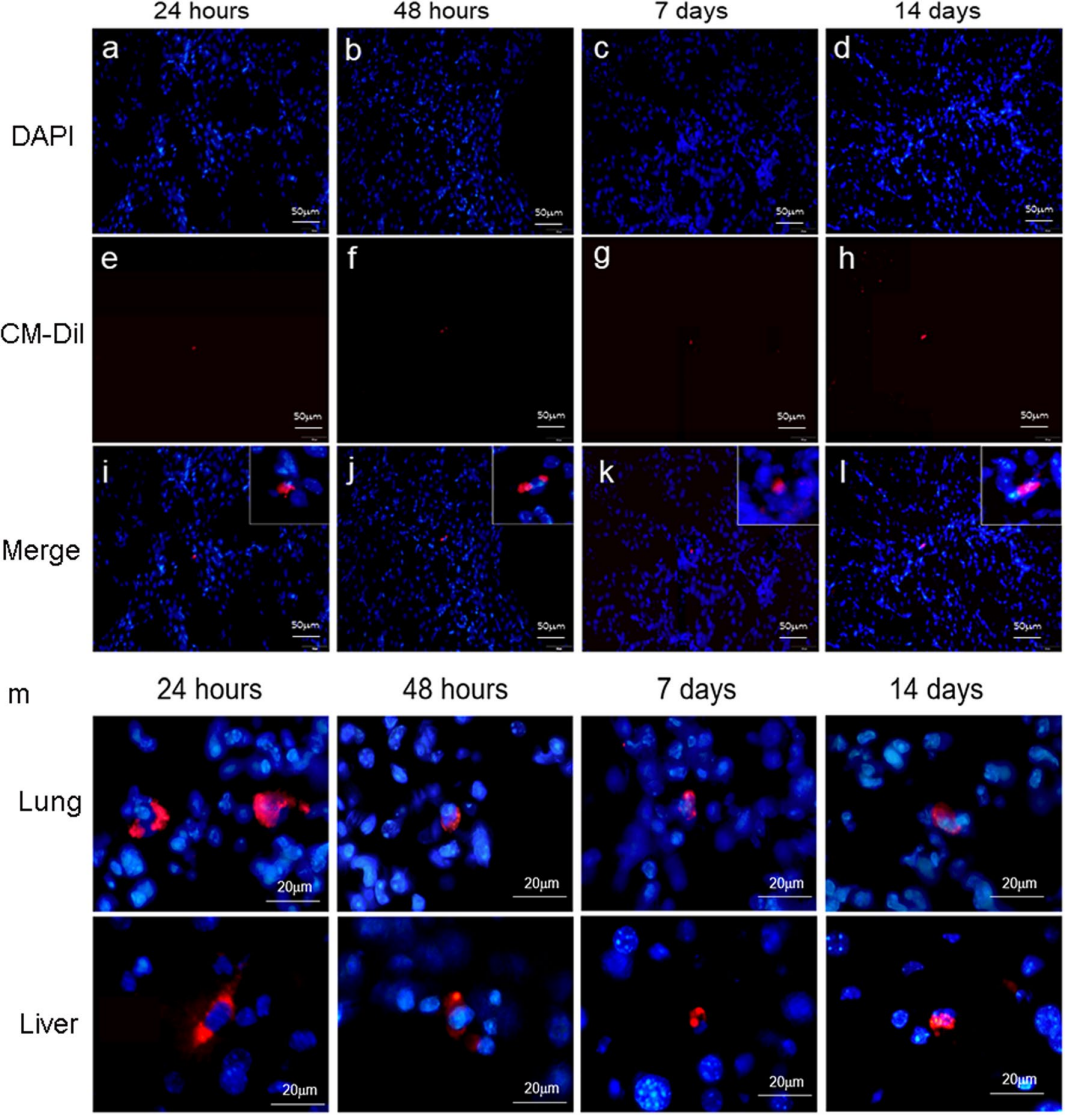
In vivo biodistribution of hUC-MSCs: **a**–**d** hUC-MSC nuclei are stained blue with DAPI, and MSCs labeled with CM-Dil show red fluorescence (**e**–**h**); **a**, **e**, **i** 24 h after the injection of hUC-MSCs; **b**, **f**, **j** 48 h after the injection of hUC-MSCs; **c**, **g**, **k** 7 days after the injection of hUC-MSCs; **d**, **h**, **l** 14 days after the injection of hUC-MSCs. Scale bar, 50 mm. **m** CM-Dil labeled hUC-MSCs are diabetic lungs and livers. Scale bar, 20 mm. Magnification, × 400

*Characterization of hUC-MSCs and *in vivo* biodistribution* Human UC-MSCs exhibited multi-lineage (adipogenic, osteogenic, and chondrogenic) differentiation capabilities (Fig. 2A–C) and demonstrated expected proportions of positive and negative staining for relevant surface markers [CD29 (99.69%), CD44 (99.79%), CD73 (98.38%), CD90 (99.90%), CD105 (99.82%), CD45 (0.69%), HLA-DR (0.46%)] by flow cytometry (Fig. 2D). Following single i.v. injections of CM-Dil-labelled hUC-MSCs in DM mice, fluorescent cells were detected in the livers, lungs, and kidneys 24 h, 48 h, 7 days and 14 days later (Fig. 3). At the later time-points, numbers of fluorescent cells detected in kidney and other organs were very low, precluding a formal quantitative analysis. These observations indicate that CM-Dil^+^ hUC-MSCs persisted in the kidneys as well as in the liver and lungs for up to 2 weeks in the setting of DM, albeit at very low frequency.

**Fig. 4:**
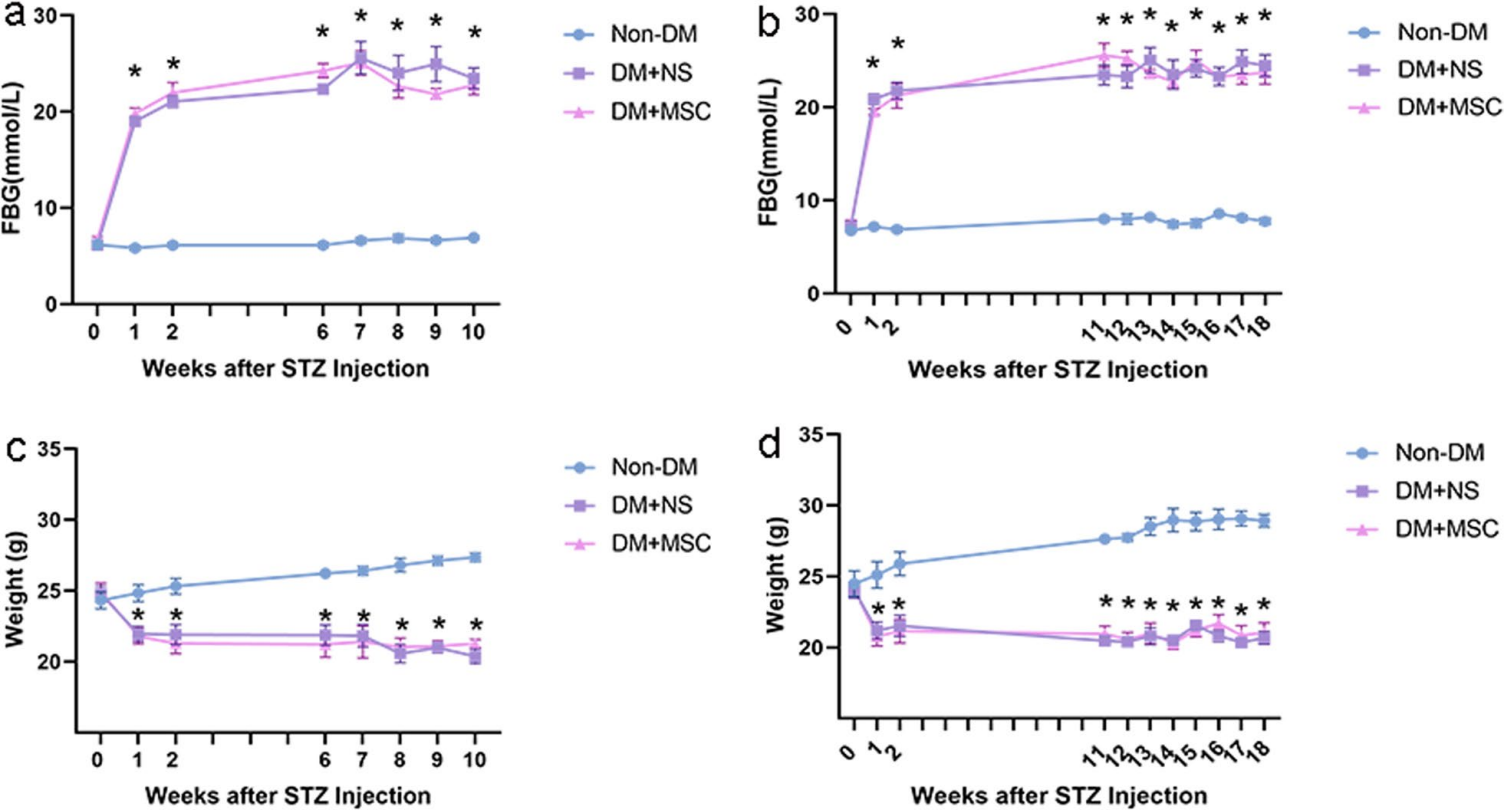
Monitoring of diabetic and non-diabetic mice from two in vivo experiments: **a**, **b**: Graphical presentations of the results of serial FBG measurements of groups of mice (n = 6 per group) from experiments of 10 weeks (**a**) and 18 weeks **b** total duration. **c**, **d**: Graphical presentations of the results of serial body weight measurements from the same two experiments. Data are displayed as mean ± SEM. *p < 0.05 versus Non-DM group

*Effects of early and later hUC-MSC injections on parameters of diabetes, renal function and kidney histology* For the two in vivo experiments of 10- and 18-weeks total duration respectively, serial FBG and body weight demonstrated significant and closely comparable trends of hyperglycemia and weight loss in male mice receiving STZ followed by single i.v. injections of hUC-MSCs or NS compared to groups of non-DM mice (Fig. 4).

In the same two experiments, urine albumin creatinine ratio (uACR) and kidney weight index were calculated at the 10 week and 18 week end-points (results summarized in Table 1). As shown, uACR was increased in the groups with DM compared to the relevant Non-DM group. However, for both experiments, uACR was lower in recipients of hUC-MSC injections than in those injected with NS. In contrast, kidney weight index, while increased in DM compared to Non-DM groups, did not differ between hUC-MSC- and NS-treated mice for either administration time-point.

**Table 1:** Urine albumin and kidney weight parameters at the end-points of two in vivo experiments

Parameters	10 weeks			18 weeks		
	Non-DM1	DM + NS1	DM + MSC1	Non-DM2	DM + NS2	DM + MSC2
UMA (mg/L)	3.580 ± 0.505	6.520 ± 1.023	5.460 ± 1.103	3.872 ± 0.778	4.961 ± 1.375	2.036 ± 0.438
Ucr (mmol/L)	5.152 ± 0.837	1.028 ± 0.178*	1.270 ± 0.267*	2.707 ± 0.171	0.499 ± 0.081*	0.277 ± 0.027*
uACR (mg/g)	8.080 ± 0.836	74.520 ± 7.334*	47.740 ± 3.616*^#^	12.860 ± 1.994	88.402 ± 3.240*	70.340 ± 17.470*^#^
KW (g)	0.200 ± 0.009	0.156 ± 0.007*	0.160 ± 0.006*	0.178 ± 0.007	0.147 ± 0.003*	0.165 ± 0.011
KI (mg/g)	6.189 ± 0.143	7.408 ± 0.217*	7.013 ± 0.240*	6.456 ± 0.196	8.007 ± 0.429*	8.045 ± 0.311*

Non-DM1 = Nondiabetic mice analyzed 10 weeks after initiation of the experiments; DM + NS1 = Diabetic mice injected with normal saline and analyzed 10 weeks after induction of diabetes; DM + MSC1: Diabetic mice injected with hUC-MSCs and analyzed 10 weeks after induction of diabetes; Non-DM2 = Nondiabetic mice analyzed 18 weeks after initiation of the experiments; DM + NS2 = Diabetic mice injected with normal saline and analyzed 18 weeks after induction of diabetes; DM + MSC2: Diabetic mice injected with hUC-MSCs and analyzed 18 weeks after induction of diabetes; UMA = Urine microalbumin concentration; Ucr = Urine creatinine concentration; uACR = Urine albumin creatinine ratio; KW = Kidney weight; KI, Kidney index. Data are presented as the mean ± SEM

* = *p* < 0.05 versus Non-DM group, ^#^ = *p* < 0.05 versus DM + NS group

**Fig. 5:**
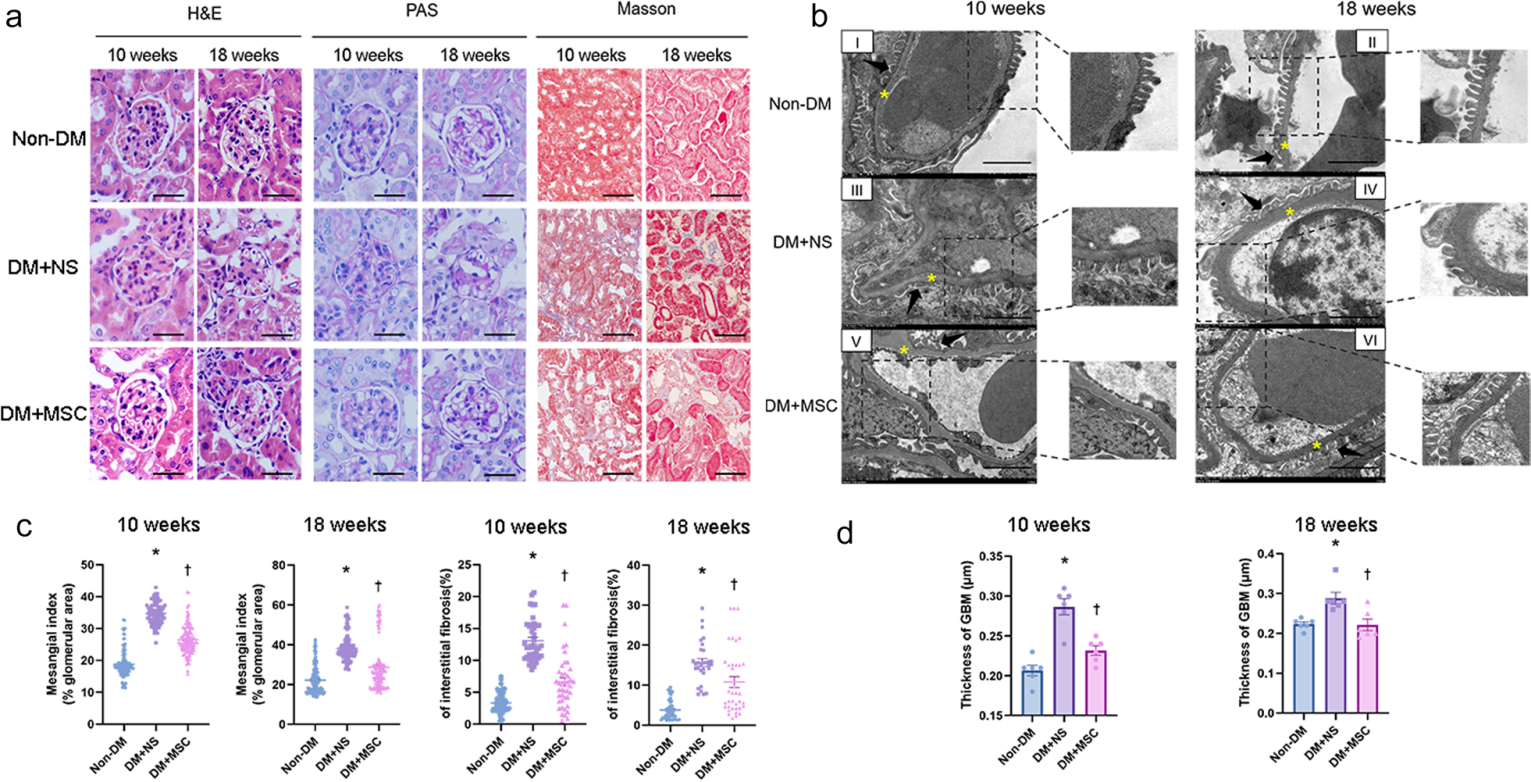
Kidney histology and ultrastructural analyses from two in vivo experiments. **a** Representative images of H&E, PAS, and Masson’s trichrome stained kidney sections from Non-DM, DM + NS and DM + MSC mice, analyzed 10 and 18 weeks after induction of DM [magnification: 400× (Scale bar 36  μm) and 200× (Scale bar 76  μm)]. **b** Graphical representation of quantification of Mesangial index (% glomerular area) and tubulointerstitial fibrosis of Non-DM, DM + NS and DM + MSC mice at 10 and 18 weeks after induction of DM. **c** Representative examples of transmission electron microscopy images of glomeruli of individual animals from each of experimental groups demonstrating ultrastructural features of the GBM and podocytes (Scale bar 2 μm). Arrowhead represents foot processes of the podocytes; asterisk represents GBM. **d** Graphical representation of quantification of thickness of GBM. For graphs, data are presented as mean ± SEM. *p < 0.05 versus Non-DM group. ^†^*p* < 0.05 versus DM + NS group

**Fig. 6:**
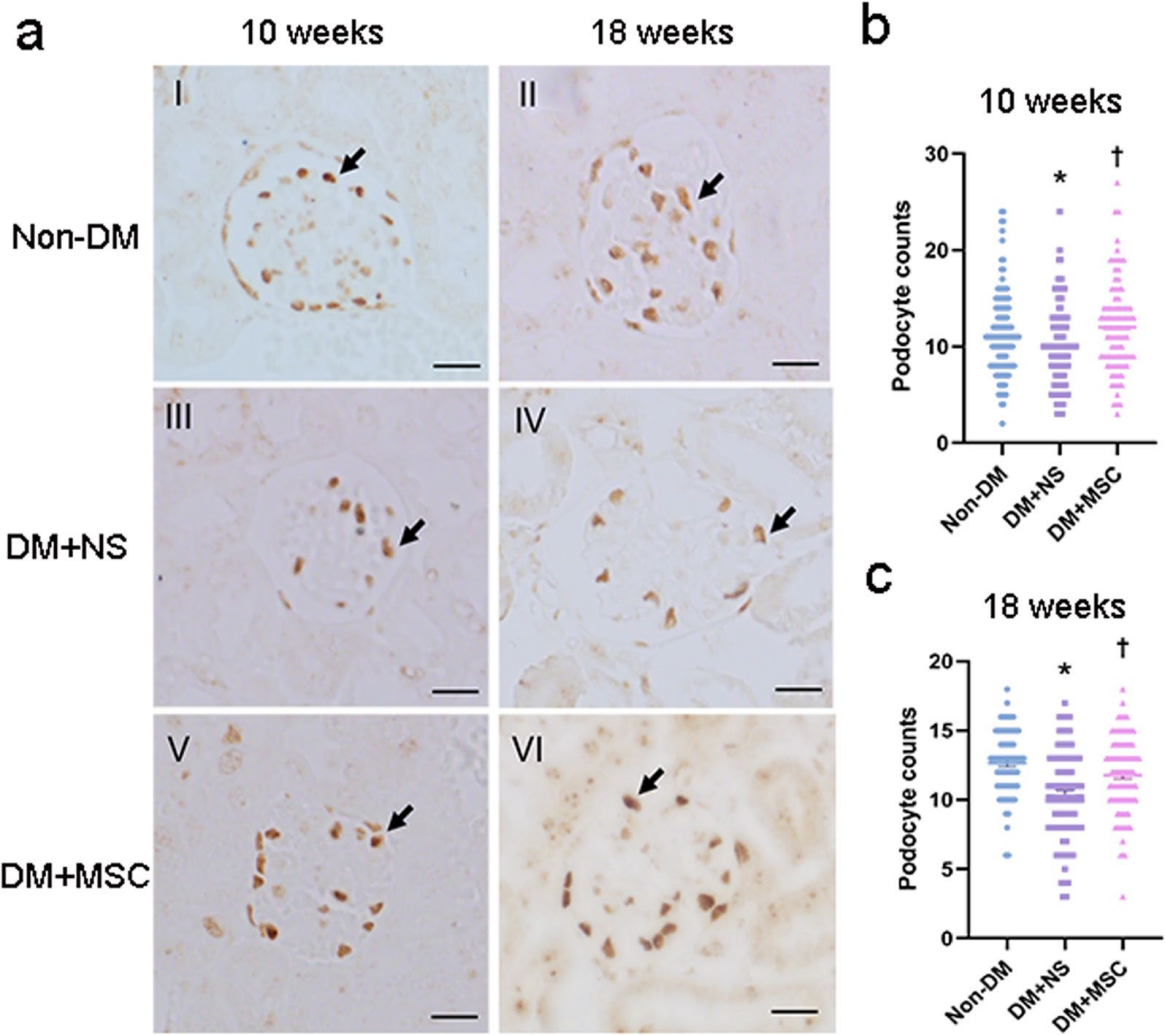
Immunohistochemical analysis of podocyte numbers at the end-points of two in vivo experiments: **a** The representative images of immunohistochemistry for WT-1 (arrowheads) in kidney sections from groups of Non-DM, DM + NS and DM + MSC mice, analyzed 10 weeks and 18 weeks after induction of DM (Scale bar 38 μm). **b**, **c** Graphical representations of podocyte counts per glomerular cross section at the two time points in Non-DM, DM + NS, and DM + MSC groups. Data are presented as mean ± SEM, n = 6 per group. *p < 0.05 versus Non-DM group. ^†^*p* < 0.05 versus DM + NS group

For each of the three groups from the two in vivo experiments, representative examples of glomeruli from H&E and PAS-stained kidney sections and tubulointerstitial regions from MT-stained sections are shown in Fig. 5a. From these, mesangial index and % interstitial fibrosis was derived for each kidney by blinded image analysis. As shown in Fig. 5b, kidneys of diabetic animals at both early and later time-points demonstrated mesangial expansion and increased interstitial fibrosis that were reduced in hUC-MSC groups compared to NS-injected groups. In the same experiments, ultrastructural features consistent with DN—increased GBM thickness and podocyte foot process effacement—were demonstrated by TEM in DM (Fig. 5c). Quantitative analysis of GBM thickness demonstrated increases in NS-injected DM compared to Non-DM mice which was reduced in mice that had received hUC-MSC injections at both early and later time-points (Fig. 5d). Finally, the effects of DM and hUC-MSC administration on podocyte numbers were investigated by immunohistochemistry of kidney sections for WT-1. As shown in Fig. 6, the number of podocytes per glomerular section was lower in NS-treated diabetic mice compared to Non-DM mice but was preserved in diabetic mice that had received single injections of hUC-MSCs at both early and later time-points. It was concluded from these results that hUC-MSC injections had no influence on the severity of STZ-induced DM but resulted in reduced albuminuria, amelioration of glomerular and interstitial abnormalities and prevention of podocyte loss following either early or later administration.

**Fig. 7:**
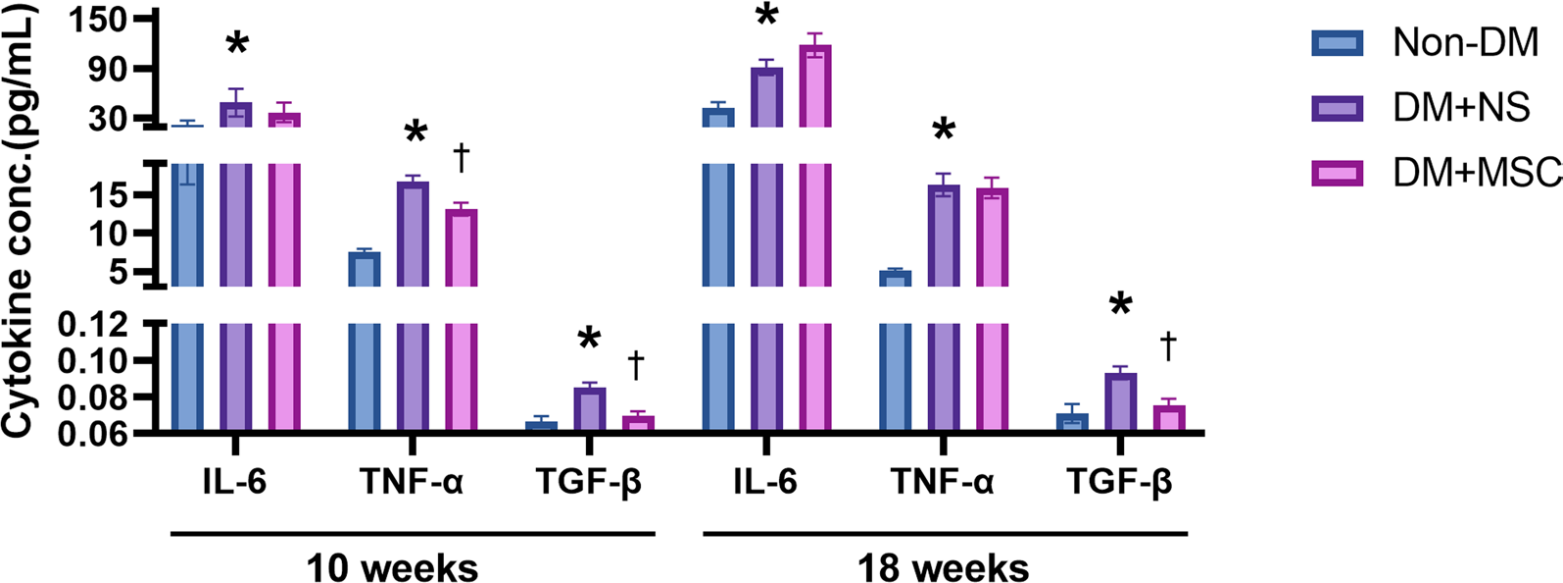
Serum concentrations of pro-inflammatory and pro-fibrotic cytokines at the end-points of two in vivo experiments: Graphical representations of the concentrations of IL-6, TNF-α and TGF-β measured by ELISA in serum samples from groups of Non-DM, DM + NS and DM + MSC mice, analyzed 10 weeks and 18 weeks after induction of DM. Data are presented as mean ± SEM, n = 6 per group. ^*^p < 0.05 versus Non-DM group. ^†^*p* < 0.05 versus DM + NS group

*Effects of early and later hUC-MSC injections on systemic levels of pro-inflammatory and pro-fibrotic cytokines* Serum concentrations of the pro-inflammatory cytokines IL-6 and TNF-α and the pro-fibrotic cytokine TGF-β1 were compared for Non-DM, DM + NS and DM + hUC-MSC groups for the in vivo experiments of 10 weeks and 18 weeks duration (Fig. 7). As shown, the serum concentrations of all 3 cytokines were higher in NS-treated diabetic mice than in DM mice at both early and later time-points. In the case of IL-6 and TNF-α, hUC-MSC administration at the earlier time-point was associated with small but significant reductions compared to NS administration but this effect was not observed following administration of hUC-MSC at a later time-point. In contrast, the serum concentrations of TGF-β1 were markedly lower in diabetic recipients of hUC-MSCs compared to NS recipients at both time-points studied. These results suggest that systemic anti-inflammatory effects of single hUC-MSC injections were modest and limited to the earlier stage of DM, while systemic effects on an archetypal pro-fibrotic mediator were relatively greater and could also be observed at a later disease stage.

**Fig. 8:**
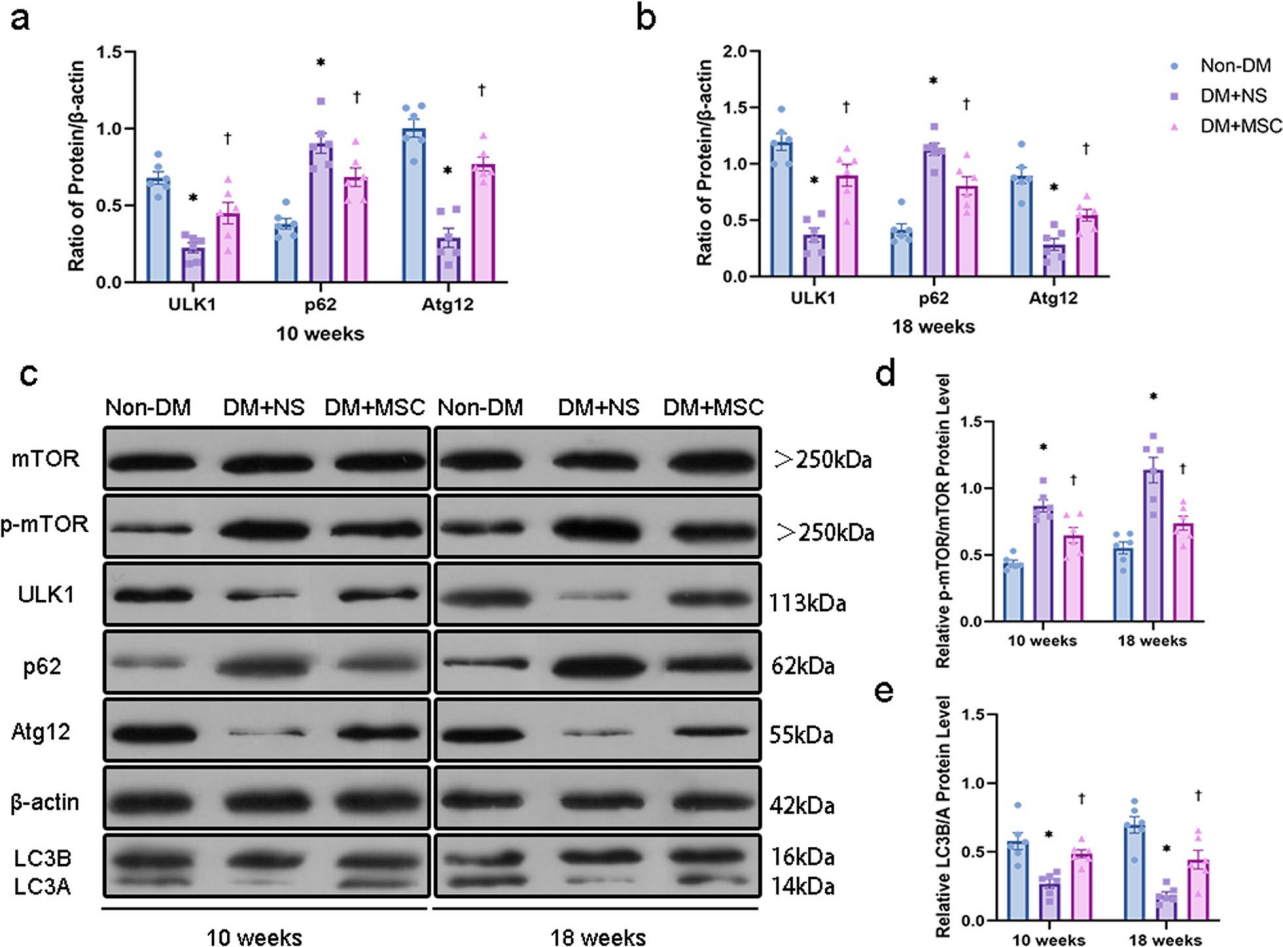
Western blots of autophagy-related proteins in whole kidney lysates at the end-points of two in vivo experiments: Representative bands (left upper panels) and graphical representations of the densitometric quantification of autophagy-related proteins (mTOR, p-mTOR, ULK1, Atg12, p62, LC3A/B) and a housekeeping protein (β-actin) from Western blots of whole kidney lysates from groups of Non-DM, DM + NS and DM + MSC mice. Full-length blots were presented in Additional file 3: Fig. S1. **a** Graphs show densitometric analysis of protein levels normalized to the β-actin expression level at 10 weeks. **b** Graphs show densitometric analysis of protein levels normalized to the β-actin expression level at 18 weeks. **c** Images are representative at 10 weeks and 18 weeks. **d** Graphs show densitometric analysis of p-mTOR protein levels normalized to the mTOR expression level. **e** Graphs show densitometric analysis of LC3B protein levels normalized to the LC3A expression level. Data are means ± SEM. *p < 0.05 versus Non-DM group. ^†^*p* < 0.05 versus DM + NS group. n = 6 biologically independent experiments

*Effects of early and later UC-MSC injections on intra-renal expression of autophagy-related proteins* Given the reported role of dysregulated autophagy in DN [23], quantification of a panel of autophagy-related proteins was carried out by Western blotting of kidney tissue lysates from Non-DM, DM + NS and DM + MSC groups from the two in vivo experiments (Fig. 8). At both 10-week and 18-week time-points, these studies demonstrated increased ratios of p-mTOR to total mTOR, increased abundance of p62, reduced abundance of ULK1 and Atg12 and reduced ratio of LC3B to LC3A in NS-DM compared to Non-DM—consistent with higher mTOR pathway activity and reduced autophagy within the kidneys of diabetic animals. Strikingly, in kidneys of diabetic mice that received single hUC-MSC injections at early and later time-points, these changes were largely reversed. These results supported a conclusion that single injections of hUC-MSCs were associated with correction of mTOR-mediated inhibition of autophagy within renal cells whether administered early or later in the course of DN.

## Discussion

Diabetic nephropathy is the most common cause of CKD and affects high proportions of people with both type 1 and type 2 diabetes. Kidney damage caused by long-term hyperglycemia causes insufficient clearance of metabolic waste and excess fluid in the body, which ultimately leads to secondary hypertension and aggravates kidney damage [24–26]. At present, the drugs prescribed for the treatment of DN are mainly used to control blood glucose, reduce blood pressure and regulate glomerular hemodynamics [27]. However, even the most recently-introduced drug regimens effectively delay but do cannot fully halt or reverse the progression of DN. Multi-drug treatment may also be accompanied by serious adverse reactions, and drug compliance may be challenging for some. In this context, pre-clinical research demonstrating reproducible beneficial effects of systemic MSCs in diverse models of diabetes and DN has offered strong evidence for the application of disease-modulating regenerative cell therapies to patients with DKD. Nonetheless, some important parameters for successful clinical translation remain poorly understood [28–30]. While the limitations of a xenogeneic experimental model (human cells into immunocompetent mice), including the potential for cross-species incompatibilities [31], must be acknowledged, we selected this model on the basis that pre-clinical models in which the human cell product intended for eventual clinical use is administered to animal recipients are known to provide valuable pre-clinical insights and are preferable, if not essential for regulatory approval of a clinical trial protocol. In this study, fluorescent labelling was first performed to trace the bio-distribution of single i.v. injections of hUC-MSCs in immunocompetent mice with established diabetes. Interestingly, we observed that hUC-MSCs persisted for at least 14 days in multiple organs, including the kidneys. In general, our observations on biodistribution of hUC-MSCs following tail vein administration are consistent with those reported in other tracking studies in which the majority of cells localized to the lungs and liver and a minority subsequently migrated to other organs including kidneys, spleen and heart (but not brain) [32–34]. Although this could suggest that localized paracrine effects of systemically administered MSCs may be more protracted in a diabetic environment than previously assumed, it should be acknowledged that the low numbers detected within the kidneys at the later time-points cast doubt on whether localized effects play a major role in the disease modulating properties of the cells. Nonetheless, continued release of pro-repair paracrine factors capable of distributing over relatively wide areas of tissue or within the circulation could represent a distinct therapeutic feature of stem and progenitor cell-derived therapies. For example, there is growing evidence that extracellular vesicles (EVs) derived from stem cells can transport exogenous miRNAs to recipient cells in diseased kidneys [35, 36]. Furthermore, it has reported that EVs derived from adipose stem cells alleviated podocyte epithelial–mesenchymal transition under high glucose conditions [37]. From a safety perspective, our results raise no specific concerns about the potential for long-term engrafted hUC-MSCs to mediate harmful effects such as organ fibrosis, ectopic bone or cartilage formation or tumorigenesis. We also note that such concerns have not arisen, to date, from the many well-conducted clinical trials of UC-MSCs and MSCs from other tissue sources in thousands of human subjects [38].

For our pre-clinical interventional study, we elected to induce diabetes by intraperitoneal injection of STZ for 5 consecutive days in male C57BL/6 mice—a model that involves pancreatic islet destruction and, thus, has an acute onset and physiological features that more closely resembles type 1 than type 2 diabetes in humans [17, 39, 40]. As expected in male mice of this genetic strain, the STZ model resulted in profound, lasting hyperglycemia accompanied by weight loss and biochemical and micro-anatomical abnormalities that replicate the features of early-stage human DN [41, 42]. Our experimental design allowed us to compare the progression of DN changes and the effects of single dose hUC-MSC administration on both glycemic control and DN parameters following 10 week and 18 weeks periods of established diabetes. Importantly, in both cases, we observed no influence of hUC-MSCs on glycaemia and weight loss—a finding that is in contrast to some previously published studies in which MSCs were administered to animals with shorter duration of STZ-induced diabetes. For instance, in a recent study in rats, Yousef et al. [43] administered MSCs 1 or 4 weeks after STZ injection, resulting in improved β islet cell function and glycaemia 7 days later. Similarly, Si et al. [44] reported increased insulin and C-peptide production and decreased blood glucose levels 2 weeks after MSC injections in diabetic rats in which STZ had been administered 1 or 3 weeks earlier. Taken together, our results and those of previously-reported studies suggest that systemic MSC therapy may improve glycaemia in type 1-like diabetes when administered soon after islet injury but do not do so at more clinically relevant later time-points [45]. A broader review of pre-clinical studies, indicates that time of MSC administration has varied widely from 25 days to 24 weeks post-STZ administration [46–48]. The results from our study provide further clarity that, for established diabetes due to β islet cell destruction, the beneficial effects of human MSC administration on kidney structure and function cannot be ascribed to secondary effects of improved glycemic control. Whether this conclusion also applies to type 2 diabetes, in which insulin resistance is the predominant initial mechanisms of hyperglycemia, cannot be determined from this study but it is notable that single i.v. infusions of allogeneic mesenchymal precursor cells did not influence glycemic control in an early-phase, randomized controlled clinical trial involving 30 adults with CKD due to type 2 diabetes.

Mildly increased urine albumin excretion (microalbuminuria) is typically observed at an early stage of DN in human type 1 diabetes and was, as expected, evident in mice with STZ-induced diabetes at both time-points studied. From a clinical perspective, microalbuminuria represents a strong predictor of subsequent overt proteinuria, reduced GFR and, eventually, end-stage kidney disease [49, 50]. The primary initial pathological features of DN include mesangial expansion, GBM thickening, and podocyte loss [51]—each of which was also observed at both 10 and 18 week time-points of the STZ-induced model in our study. The results we report here provide compelling evidence that single i.v. doses of hUC-MSCs exerted substantial modulatory effects on these cardinal pathological features of DN within the glomeruli over a 2-week post-administration time-period whether diabetes had been established for shorter or longer periods of time. These effects were accompanied by the expected reductions in urine ACR. Although validation of the lasting benefit of DN modulation in its earlier stages by i.v. hUC-MSCs will require studies of single- or multiple-dose regimens in more progressive models of DN, we believe that our results favor a cell therapy approach to stabilizing the course of kidney disease from its earliest clinic-pathological manifestations. Applied in this manner, MSC-based therapies could potentially complement the benefits of established drug therapies while avoiding the escalation of polypharmacy that typically occurs as DN progresses.

In recent decades, numerous researchers have contributed to the elucidation of molecular mechanisms of the development of DN, including a range of cell response pathways that lead to chronic inflammation and fibrosis [52, 53]. Inflammation is a key pathologic finding in chronic kidney diseases [54], including DN. Injuries to the tubules, in turn, may provoke inflammatory responses and result in renal fibrosis. The extent of damage to the kidney tubules and subsequent fibrosis due to inflammation is strongly associated with reduced GFR and with its alleviation by MSC therapies [55]. In the current study, we showed that diabetes mellitus, in this model, was associated with increased circulating cytokines, enhanced interstitial fibrosis and disordered autophagy all of which are beneficially impacted by MSC administration. While promising from a mechanistic perspective, our results to date do not prove that the amelioration of dysregulated autophagy, inflammation and fibrosis by i.v. administration of hUC-MSC are causatively linked. They do, however, provide a clear set of experimental conditions in which to perform further mechanistic studies. In the current study, we addressed systemic inflammation and the pro-fibrotic milieu by quantifying serum levels of relevant cytokines in our experimental groups at earlier and later time-points. In keeping with expectations, serum IL-6, TNF-α, and TGF-β1 were increased in DM + NS groups at both time-points compared with the Non-DM group. Of interest, however, while hUC-MSC administration resulted in reductions of the inflammatory cytokines IL-6 and TNF-α levels at the earlier but not the later time-point, reductions in the archetypal pro-fibrotic cytokine TGF-β1 were observed at both times. Consistent with this, histological analysis demonstrated that diabetes caused increased renal interstitial fibrosis by 10 weeks, which had worsened by 18 weeks and was substantially reduced by hUC-MSC administration at both time-points. The central role of TGF-β in MSC multi-potency and therapeutic effects in a range of diseases should be emphasized. It is expressed as three isoforms (TGF-β1, TGF-β2 and TGF-β3) which mediate signaling through a common set of serine/threonine kinase receptors. The pleotropic effects of this system on cells and tissues of mesenchymal origin and on chronic inflammation represent an important target of MSC-based therapies for the complications of diabetes [56–58]. Anti-fibrotic effects of hUC-MSCs have been previously reported in models of DN [59]. The results we report here strengthen the evidence for specific anti-fibrotic mechanisms of action of systemic MSC administration in the setting of diabetes and, furthermore, suggest that they may be more therapeutically important following prolonged duration of diabetes than the well-established anti-inflammatory effects of MSC-based therapies. As our primary analyses were performed 2 weeks after hUC-MSC administration, we cannot determine the full duration of anti-fibrotic effects and whether repeated dosing may be necessary to maintain therapeutic benefits over a prolonged period. In subsequent experiments, we will extend the observation time and investigate multi-dose regimens.

The role of immunomodulatory effects in our experimental model and in vivo results also merits consideration. As is well documented, hUC-MSCs have a range of potential immunomodulatory properties, interacting directly or indirectly with cells of both the innate and adaptive arms of the immune system [60]. In addition to representing an important aspect of their mechanism of disease modulation for conditions with dysregulated immune/inflammatory responses, the immunomodulatory effects of UC-MSCs have also been suggested to minimize the risk of initiating an allogeneic immune response when administered in vivo [61]. This property, as well as their ease of collection, has formed the basis of an increasing interest in the use of allogeneic UC-MSCs in both pre-clinical studies and clinical trials [62, 63]. These properties were also central to our selection of hUC-MSCs as the therapeutic intervention in a fully immunocompetent mouse (xenogeneic) model of diabetes/diabetic nephropathy. Specifically, we anticipated that the immunomodulatory properties of hUC-MSCs would prevent their immediate removal, lack of efficacy or adverse inflammatory effects mediated by xenogeneic immune responses. Our results indicate that this was likely the case as no adverse effects or increased systemic inflammation occurred among experimental animals after cell injection, while multiple significant disease modulating effects were observed. Furthermore, biodistribution studies indicated persistence of small numbers of cells in multiple organs for as long as 14 days and multiple disease modulating effects of hUC-MSCs. We also hypothesized that hUC-MSCs would mediate immunomodulatory effects in the experimental animals as part of a mechanism of action to slow the progression of kidney injury due to diabetic nephropathy. Although we found some evidence of this, the effects on fibrosis and on expression of autophagy-related proteins in the kidney were more striking.

Autophagy is an adaptive or "programmed cell survival" mechanism that protects the organism when cells are exposed to external injurious stimuli to maintain cellular homeostasis. As a central element for signaling cell growth and enhancing protein translation, mammalian target of rapamycin (mTOR), when inhibited, induces autophagy. Likewise, as a critical feedback mechanism, reactivation of mTOR terminates autophagy and initiates lysosome reformation [64]. Studies have shown that abnormal autophagy is closely related to the pathogenesis of DN [15, 65]. It should be noted that autophagy is a multifactorial process which not only includes the formation of autophagosomes but a dynamic flux that involves the degradation upon fusion with lysosomes. While, we have demonstrated that increased mTOR activity and suppressed autophagy within the kidneys were induced by diabetes mellitus and restored by MSC administration, we have not yet experimentally evaluated whether the observed effects on autophagy were related to the accumulation of autophagosomes as a result of induced autophagic activity or accumulation due to ineffective lysosomal clearance. Nonetheless, the results we report for modulation of intra-renal autophagy following hUC-MSC administration at two different time-points, are consistent with those recently reported by Li et al. and lay the groundwork for future experimental dissection of the nature of dysregulated autophagy as a targetable mechanism of progression of diabetic kidney disease [66].

In our study, the glomerular and tubulointerstitial abnormalities that were observed in the DM + NS group at both time-points were associated with evidence of deficits in autophagy within the kidneys. Most notably, we demonstrated reduced abundance of the autophagy markers ULK1 and Atg12 as well as a marked upregulation of mTOR pathway activity in renal tissue of NS-treated DM mice compared with those of Non-DM controls—effects that were reversed by hUC-MSC administration. Our results for p62 and LC3B/A were also consistent with decreased intra-renal autophagy following 10 and 18 weeks of diabetes and with substantial reversal of this effect by single i.v. administrations of hUC-MSCs. Overall, these findings were consistent with those of Gödel et al. [67], who reported significant upregulation of mTOR in human patients with progressive DN. These authors also demonstrated that activation of mTORC1 inhibits diabetes-related autophagy in renal tissues of DM mice and patients. In line with these results, Lenoir et al. [68] evaluated the role of autophagy in the endothelial cells of diabetic kidneys using endothelial cell-specific Atg5-deficient mice. In this study, ultrastructural analysis revealed glomerular endothelial cell cytoplasmic disorganization and vacuolization, as well as detached cells—most likely endothelial cells—in the lumen of the glomerular capillaries of diabetic Atg5-deficient mice. Our mechanistic findings with hUC-MSC injections are also in keeping with recent reports of the effects of rodent MSCs and MSC-derived soluble products or EVs to suppress TGF-β1 and mTOR activity and increase autophagy in whole kidneys and cultured podocytes under acute kidney injury and diabetic conditions [69–71]. Our results do not yet reveal the mechanism or mechanisms whereby intravenously delivered hUC-MSCs restore a more physiological level of autophagy within the kidneys at the time-points studied and this will be the focus of future experimental work. A paracrine mechanism may be most likely as hUC-MSCs may be induced to release a variety of growth factors, cytokines and EVs containing microRNAs and proteins, which have potential to promote autophagy and enhance cellular repair and survival in recipient cells. For example, Zhang et al. showed that UC-MSC-derived EVs contained many different microRNAs (miRNAs), among which miR-20a could alleviate the abnormal expression of genes related to autophagy, such as active mTOR, P62, and LC3II in liver injury [72]. Direct cell–cell interactions such as mitochondrial transfer also represent a potential mechanism for restoration of autophagy in primary renal cells [73]. However, the fact that only small numbers of hUC-MSCs were detected in the kidneys of diabetic mice between 24 and 14 days after i.v. injection, appears to argue against local intra-renal effects being the primary reno-protective mechanism.

In summary, our results demonstrate that single i.v. injections of hUC-MSCs ameliorated archetypal glomerular and interstitial structural abnormalities of DN in immunocompetent male mice following longer and shorter durations of diabetes—suggesting a relatively wide potential time-window for beneficial effects of this cell therapy in patients with early clinical evidence of diabetic kidney disease. Mechanistically, we observe that anti-fibrotic effects of systemic hUC-MSCs were more durable than anti-inflammatory effects and that single injections of hUC-MSCs resulted in correction of enhanced mTOR pathway activation and consequence quenching of autophagy within the kidneys. Future work on the potential for hUC-MSC-based therapies to be applied to the early stages of diabetic kidney disease will focus on elucidating the duration of anti-fibrotic and other beneficial effects and the mechanistic actions of single and multiple i.v. MSC doses on models of progressive DN. Given the known sexual dimorphism of diabetes and diabetic complications, it will also be important to compare effects of hUC-MSC administration in male and female mice [74].

## Conclusion

Single intravenous injections of hUC-MSCs ameliorated glomerular abnormalities and interstitial fibrosis in a male mouse model of STZ-induced diabetes without affecting hyperglycemia, whether administered at relatively short or longer duration of diabetes. At both time-points, the reno-protective effects of hUC-MSCs were associated with reduced circulating TGF-β1 and restoration of intra-renal autophagy. The study adds to a growing body of pre-clinical and early-phase human clinical data that support the potential benefits of systemic MSC therapies in patients with diabetic nephropathy [75, 76]. Specifically, they lend further strength to the body of evidence that hUC-MSCs may be a viable therapeutic option for human diabetic kidney disease. Nonetheless, further in vivo experimental studies will be needed to determine whether the findings of efficacy and the mechanistic insights reported here can be robustly translated to a clinical setting. If this proves to be the case, the eventual success of this therapy will depend on the outcome of well-designed phase 2 and 3 clinical trials.

### Supplementary Information

**Additional file 1**. Table S1 GDS.

**Additional file 2**. Supplemental methods.

**Additional file 3**. Figure S1. Full-length gels and blots of Figure 8C.

## Data Availability

The data that support the findings of this study are available from the corresponding author upon reasonable request.

## Abbreviations

DM: Diabetes mellitus
DN: Diabetic nephropathy
T1DM: Type 1 DM
T2DM: Type 2 DM
CKD: Chronic kidney disease
(hUC)-MSCs: (Human umbilical cord-derived) mesenchymal stem cells
STZ: Streptozocin
SPF: Specific pathogen free
FBG: Fasting blood glucose
GDS: General distress scoring
PBS: Phosphate-buffered saline
NS: Normal saline
(u)ACR: (Urine) albumin-creatinine ratios
TEM: Transmission electron microscopy
KI: Kidney index
KW: Kidney weight
H&E: Hematoxylin and eosin
PAS: Periodic acid-Schiff
GBM: Glomerular basement membrane
Wilm’s tumor 1: WT1
ELISA: Enzyme-linked immunosorbent assay
(IL)-6: Interleukin-6
TNF-α: Tumor necrosis factor alpha
TGF-β1: Transforming growth factor beta 1
PVDF: Polyvinylidene difluoride
EVs: Extracellular vesicles
mTOR: Mammalian target of rapamycin

## References

[1] Sun H, Saeedi P, Karuranga S, Pinkepank M, Ogurtsova K, Duncan BB (2022). IDF Diabetes Atlas: Global, regional and country-level diabetes prevalence estimates for 2021 and projections for 2045. Diabetes Res Clin Pract.

[2] Jager KJ, Kovesdy C, Langham R, Rosenberg M, Jha V, Zoccali C (2019). A single number for advocacy and communication-worldwide more than 850 million individuals have kidney diseases. Nephrol Dial Transplant.

[3] Zhang XX, Kong J, Yun K (2020). Prevalence of diabetic nephropathy among patients with type 2 diabetes mellitus in China: a meta-analysis of observational studies. J Diabetes Res.

[4] Wang J, Xiang H, Lu Y, Wu T, Ji G (2021). New progress in drugs treatment of diabetic kidney disease. Biomed Pharmacother.

[5] Kalantar-Zadeh K, Jafar TH, Nitsch D, Neuen BL, Perkovic V (2021). Chronic kidney disease. Lancet.

[6] Cockwell P, Fisher LA (2020). The global burden of chronic kidney disease. Lancet.

[7] Hofherr A, Williams J, Gan LM, Soderberg M, Hansen PBL, Woollard KJ (2022). Targeting inflammation for the treatment of diabetic kidney disease: a five-compartment mechanistic model. BMC Nephrol.

[8] Griffin TP, Martin WP, Islam N, O'Brien T, Griffin MD (2016). The promise of mesenchymal stem cell therapy for diabetic kidney disease. Curr Diab Rep.

[9] Keating A (2006). Mesenchymal stromal cells. Curr Opin Hematol.

[10] Bernardo ME, Locatelli F, Fibbe WE (2009). Mesenchymal stromal cells. Ann N Y Acad Sci.

[11] Muller L, Tunger A, Wobus M, von Bonin M, Towers R, Bornhauser M (2021). Immunomodulatory properties of mesenchymal stromal cells: an update. Front Cell Dev Biol.

[12] Mebarki M, Abadie C, Larghero J, Cras A (2021). Human umbilical cord-derived mesenchymal stem/stromal cells: a promising candidate for the development of advanced therapy medicinal products. Stem Cell Res Ther.

[13] Mebarki M, Iglicki N, Marigny C, Abadie C, Nicolet C, Churlaud G (2021). Development of a human umbilical cord-derived mesenchymal stromal cell-based advanced therapy medicinal product to treat immune and/or inflammatory diseases. Stem Cell Res Ther.

[14] Zhao Y, Zhang W, Jia Q, Feng Z, Guo J, Han X (2018). High dose vitamin E attenuates diabetic nephropathy via alleviation of autophagic stress. Front Physiol.

[15] Liu WJ, Huang WF, Ye L, Chen RH, Yang C, Wu HL (2018). The activity and role of autophagy in the pathogenesis of diabetic nephropathy. Eur Rev Med Pharmacol Sci.

[16] Zhong Y, Luo R, Liu Q, Zhu J, Lei M, Liang X (2022). Jujuboside A ameliorates high fat diet and streptozotocin induced diabetic nephropathy via suppressing oxidative stress, apoptosis, and enhancing autophagy. Food Chem Toxicol.

[17] Kim JY, Lee SH, Song EH, Park YM, Lim JY, Kim DJ (2009). A critical role of STAT1 in streptozotocin-induced diabetic liver injury in mice: controlled by ATF3. Cell Signal.

[18] Rodrigues RR, Gurung M, Li Z, Garcia-Jaramillo M, Greer R, Gaulke C (2021). Transkingdom interactions between *Lactobacilli* and hepatic mitochondria attenuate western diet-induced diabetes. Nat Commun.

[19] Practical use of distress scoring systems in the application of humane endpoints.

[20] Weir C, Morel-Kopp MC, Gill A, Tinworth K, Ladd L, Hunyor SN (2008). Mesenchymal stem cells: isolation, characterisation and in vivo fluorescent dye tracking. Heart Lung Circ.

[21] Gangadhariah MH, Luther JM, Garcia V, Paueksakon P, Zhang MZ, Hayward SW (2015). Hypertension is a major contributor to 20-hydroxyeicosatetraenoic acid-mediated kidney injury in diabetic nephropathy. J Am Soc Nephrol.

[22] Gao F, Yao M, Cao Y, Liu S, Liu Q, Duan H (2016). Valsartan ameliorates podocyte loss in diabetic mice through the Notch pathway. Int J Mol Med.

[23] Gonzalez CD, Carro Negueruela MP, Nicora Santamarina C, Resnik R, Vaccaro MI (2021). Autophagy dysregulation in diabetic kidney disease: from pathophysiology to pharmacological interventions. Cells.

[24] Tuttle KR, Bakris GL, Bilous RW, Chiang JL, de Boer IH, Goldstein-Fuchs J (2014). Diabetic kidney disease: a report from an ADA Consensus Conference. Diabetes Care.

[25] Dronavalli S, Duka I, Bakris GL (2008). The pathogenesis of diabetic nephropathy. Nat Clin Pract Endocrinol Metab.

[26] Van Buren PN, Toto R (2011). Hypertension in diabetic nephropathy: epidemiology, mechanisms, and management. Adv Chronic Kidney Dis.

[27] Keri KC, Samji NS, Blumenthal S (2018). Diabetic nephropathy: newer therapeutic perspectives. J Community Hosp Intern Med Perspect.

[28] Wang Y, Shan SK, Guo B, Li F, Zheng MH, Lei LM (2021). The multi-therapeutic role of MSCs in diabetic nephropathy. Front Endocrinol (Lausanne).

[29] Wu Y, Zhang C, Guo R, Wu D, Shi J, Li L (2021). Mesenchymal stem cells: an overview of their potential in cell-based therapy for diabetic nephropathy. Stem Cells Int.

[30] Hickson LJ, Herrmann SM, McNicholas BA, Griffin MD (2021). Progress toward the clinical application of mesenchymal stromal cells and other disease-modulating regenerative therapies: examples from the field of nephrology. Kidney360.

[31] Oloyo AK, Ambele MA, Pepper MS (2017). Contrasting views on the role of mesenchymal stromal/stem cells in tumour growth: a systematic review of experimental design. Stem Cells Biol Eng.

[32] de Witte SFH, Luk F, Sierra Parraga JM, Gargesha M, Merino A, Korevaar SS (2018). Immunomodulation by therapeutic mesenchymal stromal cells (MSC) is triggered through phagocytosis of MSC by monocytic cells. Stem Cells.

[33] Gaafar T, Attia W, Mahmoud S, Sabry D, Aziz OA, Rasheed D (2017). Cardioprotective effects of Wharton jelly derived mesenchymal stem cell transplantation in a rodent model of myocardial injury. Int J Stem Cells.

[34] Gallagher D, Siddiqui F, Fish J, Charlat M, Chaudry E, Moolla S (2019). Mesenchymal stromal cells modulate peripheral stress-induced innate immune activation indirectly limiting the emergence of neuroinflammation-driven depressive and anxiety-like behaviors. Biol Psychiatry.

[35] Phinney DG, Pittenger MF (2017). Concise review: MSC-derived exosomes for cell-free therapy. Stem Cells.

[36] Nagaishi K, Mizue Y, Chikenji T, Otani M, Nakano M, Konari N (2016). Mesenchymal stem cell therapy ameliorates diabetic nephropathy via the paracrine effect of renal trophic factors including exosomes. Sci Rep.

[37] Jin J, Wang Y, Zhao L, Zou W, Tan M, He Q (2020). Exosomal miRNA-215-5p derived from adipose-derived stem cells attenuates epithelial–mesenchymal transition of podocytes by inhibiting ZEB2. Biomed Res Int.

[38] Wang Y, Yi H, Song Y (2021). The safety of MSC therapy over the past 15 years: a meta-analysis. Stem Cell Res Ther.

[39] Lee SE, Jang JE, Kim HS, Jung MK, Ko MS, Kim MO (2019). Mesenchymal stem cells prevent the progression of diabetic nephropathy by improving mitochondrial function in tubular epithelial cells. Exp Mol Med.

[40] Tesch GH, Allen TJ (2007). Rodent models of streptozotocin-induced diabetic nephropathy. Nephrology (Carlton).

[41] Gurley SB, Clare SE, Snow KP, Hu A, Meyer TW, Coffman TM (2006). Impact of genetic background on nephropathy in diabetic mice. Am J Physiol Renal Physiol.

[42] Qi Z, Fujita H, Jin J, Davis LS, Wang Y, Fogo AB (2005). Characterization of susceptibility of inbred mouse strains to diabetic nephropathy. Diabetes.

[43] Yousef HN, Sakr SM, Sabry SA (2022). Mesenchymal stem cells ameliorate hyperglycemia in type I diabetic developing male rats. Stem Cells Int.

[44] Si Y, Zhao Y, Hao H, Liu J, Guo Y, Mu Y (2012). Infusion of mesenchymal stem cells ameliorates hyperglycemia in type 2 diabetic rats: identification of a novel role in improving insulin sensitivity. Diabetes.

[45] Packham DK, Fraser IR, Kerr PG, Segal KR (2016). Allogeneic mesenchymal precursor cells (MPC) in diabetic nephropathy: a randomized, placebo-controlled dose escalation study. EBioMedicine.

[46] Ezquer F, Giraud-Billoud M, Carpio D, Cabezas F, Conget P, Ezquer M (2015). Proregenerative microenvironment triggered by donor mesenchymal stem cells preserves renal function and structure in mice with severe diabetes mellitus. Biomed Res Int.

[47] Ezquer FE, Ezquer ME, Parrau DB, Carpio D, Yanez AJ, Conget PA (2008). Systemic administration of multipotent mesenchymal stromal cells reverts hyperglycemia and prevents nephropathy in type 1 diabetic mice. Biol Blood Marrow Transplant.

[48] Grange C, Tritta S, Tapparo M, Cedrino M, Tetta C, Camussi G (2019). Stem cell-derived extracellular vesicles inhibit and revert fibrosis progression in a mouse model of diabetic nephropathy. Sci Rep.

[49] Ruggenenti P, Remuzzi G (2006). Time to abandon microalbuminuria?. Kidney Int.

[50] Cravedi P, Remuzzi G (2013). Pathophysiology of proteinuria and its value as an outcome measure in chronic kidney disease. Br J Clin Pharmacol.

[51] He M, Li Y, Wang L, Guo B, Mei W, Zhu B (2020). MYDGF attenuates podocyte injury and proteinuria by activating Akt/BAD signal pathway in mice with diabetic kidney disease. Diabetologia.

[52] Hanouneh M, Echouffo Tcheugui JB, Jaar BG (2021). Recent advances in diabetic kidney disease. BMC Med.

[53] Thomas MC (2021). Targeting the pathobiology of diabetic kidney disease. Adv Chronic Kidney Dis.

[54] Anders HJ, Schaefer L (2014). Beyond tissue injury-damage-associated molecular patterns, toll-like receptors, and inflammasomes also drive regeneration and fibrosis. J Am Soc Nephrol.

[55] Yuan Y, Yuan L, Li L, Liu F, Liu J, Chen Y (2021). Mitochondrial transfer from mesenchymal stem cells to macrophages restricts inflammation and alleviates kidney injury in diabetic nephropathy mice via PGC-1alpha activation. Stem Cells.

[56] Ehnert S, Baur J, Schmitt A, Neumaier M, Lucke M, Dooley S (2010). TGF-beta1 as possible link between loss of bone mineral density and chronic inflammation. PLoS ONE.

[57] Ehnert S, Sreekumar V, Aspera-Werz RH, Sajadian SO, Wintermeyer E, Sandmann GH (2017). TGF-beta(1) impairs mechanosensation of human osteoblasts via HDAC6-mediated shortening and distortion of primary cilia. J Mol Med (Berl).

[58] Aspera-Werz RH, Chen T, Ehnert S, Zhu S, Frohlich T, Nussler AK (2019). Cigarette smoke induces the risk of metabolic bone diseases: transforming growth factor beta signaling impairment via dysfunctional primary cilia affects migration, proliferation, and differentiation of human mesenchymal stem cells. Int J Mol Sci.

[59] Li H, Rong P, Ma X, Nie W, Chen Y, Zhang J (2020). Mouse umbilical cord mesenchymal stem cell paracrine alleviates renal fibrosis in diabetic nephropathy by reducing myofibroblast transdifferentiation and cell proliferation and upregulating MMPs in mesangial cells. J Diabetes Res.

[60] Mebarki M, Abadie C, Larghero J, Cras A (2021). Human umbilical cord-derived mesenchymal stem/stromal cells: a promising candidate for the development of advanced therapy medicinal products. Stem Cell Res Ther.

[61] Lee M, Jeong SY, Ha J, Kim M, Jin HJ, Kwon S-J (2014). Low immunogenicity of allogeneic human umbilical cord blood-derived mesenchymal stem cells in vitro and in vivo. Biochem Biophys Res Commun.

[62] Yang S, Wei Y, Sun R, Lu W, Lv H, Xiao X (2020). Umbilical cord blood-derived mesenchymal stromal cells promote myeloid-derived suppressor cell proliferation by secreting HLA-G to reduce acute graft-versus-host disease after hematopoietic stem cell transplantation. Cytotherapy.

[63] Galderisi U, Peluso G, Di Bernardo G (2021). Clinical trials based on mesenchymal stromal cells are exponentially increasing: Where are we in recent years?. Stem Cell Rev Rep.

[64] Kang R, Zeh HJ, Lotze MT, Tang D (2011). The Beclin 1 network regulates autophagy and apoptosis. Cell Death Differ.

[65] Ge X, Wang L, Fei A, Ye S, Zhang Q (2022). Research progress on the relationship between autophagy and chronic complications of diabetes. Front Physiol.

[66] Li X, Guo L, Chen J, Liang H, Liu Y, Chen W (2023). Intravenous injection of human umbilical cord-derived mesenchymal stem cells ameliorates not only blood glucose but also nephrotic complication of diabetic rats through autophagy-mediated anti-senescent mechanism. Stem Cell Res Ther.

[67] Godel M, Hartleben B, Herbach N, Liu S, Zschiedrich S, Lu S (2011). Role of mTOR in podocyte function and diabetic nephropathy in humans and mice. J Clin Invest.

[68] Lenoir O, Jasiek M, Henique C, Guyonnet L, Hartleben B, Bork T (2015). Endothelial cell and podocyte autophagy synergistically protect from diabetes-induced glomerulosclerosis. Autophagy.

[69] Ebrahim N, Ahmed IA, Hussien NI, Dessouky AA, Farid AS, Elshazly AM (2018). Mesenchymal stem cell-derived exosomes ameliorated diabetic nephropathy by autophagy induction through the mTOR signaling pathway. Cells.

[70] Jin J, Shi Y, Gong J, Zhao L, Li Y, He Q (2019). Exosome secreted from adipose-derived stem cells attenuates diabetic nephropathy by promoting autophagy flux and inhibiting apoptosis in podocyte. Stem Cell Res Ther.

[71] Tseng WC, Lee PY, Tsai MT, Chang FP, Chen NJ, Chien CT (2021). Hypoxic mesenchymal stem cells ameliorate acute kidney ischemia-reperfusion injury via enhancing renal tubular autophagy. Stem Cell Res Ther.

[72] Zhang L, Song Y, Chen L, Li D, Feng H, Lu Z (2020). MiR-20a-containing exosomes from umbilical cord mesenchymal stem cells alleviates liver ischemia/reperfusion injury. J Cell Physiol.

[73] Chen J, Wang Q, Feng X, Zhang Z, Geng L, Xu T (2016). Umbilical cord-derived mesenchymal stem cells suppress autophagy of T cells in patients with systemic lupus erythematosus via transfer of mitochondria. Stem Cells Int.

[74] Piani F, Melena I, Tommerdahl KL, Nokoff N, Nelson RG, Pavkov ME (2021). Sex-related differences in diabetic kidney disease: a review on the mechanisms and potential therapeutic implications. J Diabet Complic.

[75] Xu N, Liu J, Li X (2022). Therapeutic role of mesenchymal stem cells (MSCs) in diabetic kidney disease (DKD). Endocr J.

[76] Perico N, Remuzzi G, Griffin MD (2023). Safety and preliminary efficacy of ORBCEL-M cell therapy in diabetic kidney disease: the multicenter, randomized, placebo-controlled NEPHSTROM trial. J Am Soc Nephrol.

