# Supplementary material for: Amelioration of diabetic nephropathy in mice by a single intravenous injection of human mesenchymal stromal cells at early and later disease stages is associated with restoration of autophagy
Source: Stem Cell Res Ther. 2024 Mar 5;15:66. doi: 10.1186/s13287-024-03647-x (PMC10916232; doi:10.1186/s13287-024-03647-x)
Supplement: Supplementary file 1 — Additional file 1. Table S1 GDS. [file 13287_2024_3647_MOESM1_ESM.docx]

| Supplementary Table 1. GDS in mice | | | | | | | | | |
| --- | --- | --- | --- | --- | --- | --- | --- | --- | --- |
| Time point | After STZ injection | | Group | GDS | | | | | |
| 8-week | 8 wks | Non-DM1 | | 0 | 0 | 0 | 0 | 0 | 0 |
|  |  | DM+NS1 | | 6 | 7 | 5 | 5 | 6 | 5 |
|  |  | DM+MSC1 | | 5 | 5 | 5 | 6 | 6 | 5 |
|  | 9 wks | Non-DM1 | | 0 | 0 | 0 | 0 | 0 | 0 |
|  |  | DM+NS1 | | 6 | 7 | 6 | 6 | 7 | 6 |
|  |  | DM+MSC1 | | 4 | 5 | 4 | 4 | 4 | 6 |
|  | 10 wks | Non-DM1 | | 0 | 0 | 0 | 0 | 0 | 0 |
|  |  | DM+NS1 | | 7 | 7 | 7 | 7 | 7 | 6 |
|  |  | DM+MSC1 | | 5 | 5 | 5 | 6 | 5 | 5 |
| 16-week | 16wks | Non-DM2 | | 0 | 0 | 0 | 0 | 0 | 0 |
|  |  | DM+NS2 | | 7 | 6 | 7 | 7 | 8 | 8 |
|  |  | DM+MSC2 | | 6 | 7 | 7 | 7 | 7 | 7 |
|  | 17wks | Non-DM2 | | 0 | 0 | 0 | 0 | 0 | 0 |
|  |  | DM+NS2 | | 7 | 6 | 8 | 8 | 9 | 8 |
|  |  | DM+MSC2 | | 6 | 6 | 7 | 8 | 6 | 6 |
|  | 18wks | Non-DM2 | | 0 | 0 | 0 | 0 | 0 | 0 |
|  |  | DM+NS2 | | 8 | 7 | 8 | 8 | 9 | 8 |
|  |  | DM+MSC2 | | 7 | 6 | 7 | 8 | 7 | 7 |

**Supplementary Table 1**: General distress scoring (GDS) at the end-points of two in vivo experiments.

Abbreviations: Non-DM1 = Nondiabetic mice analyzed 10 weeks after initiation of the experiments; DM+NS1 = Diabetic mice injected with normal saline and analyzed 10 weeks after induction of diabetes; DM+MSC1: Diabetic mice injected with hUC-MSCs and analyzed 10 weeks after induction of diabetes; Non-DM2 = Nondiabetic mice analyzed 18 weeks after initiation of the experiments; DM+NS2 = Diabetic mice injected with normal saline and analyzed 18 weeks after induction of diabetes; DM+MSC2: Diabetic mice injected with hUC-MSCs and analyzed 18 weeks after induction of diabetes.
