# Supplementary material for: Amelioration of diabetic nephropathy in mice by a single intravenous injection of human mesenchymal stromal cells at early and later disease stages is associated with restoration of autophagy
Source: Stem Cell Res Ther. 2024 Mar 5;15:66. doi: 10.1186/s13287-024-03647-x (PMC10916232; doi:10.1186/s13287-024-03647-x)
Supplement: Supplementary file 2 — Additional file 2. Supplemental methods. [file 13287_2024_3647_MOESM2_ESM.docx]

## METHOD supplements

Kidneys (n=18) were dissected and post-fixed in 4% paraformaldehyde solution, cryoprotected in a sucrose gradient (10-30% in 0.2M phosphate buffer). Longitudinal sections were used to study the structure of kidney.

## *H&E staining*

Hematoxylin-eosin (HE) Staining Kit was obtained from Solarbio (G1120, Beijing, China). The processed paraffin sections taken at 3-4 micrometres were rinsed with PBS for 3 times and then placed in the hematoxylin for 10min. The sections were rinsed with running water for 4min and directly placed in alcohol hydrochloride for 3s without moisture. The sections were rinsed again with running water for 4min and then placed in eosin solution for staining for about 5min. After rinsing with tap water for 4min staining was observed under the microscope for quality control. Following this the slides are dehydrated and cleared by placing in 70% ethanol for 5min, 80% ethanol for 5min, 90% ethanol for 5min, 100% ethanol for 5min, 1/2 anhydrous ethanol +1/2 xylene for 10 min, xylene I for 10 min, xylene II for 10 min. Neutral gum is used for mounting without air bubbles. The slides are allowed dry at this point prior to analysis. Photos were taken using an Olympus B53 microscope and camera system with Cellsens software (V2.1, Olympus, Japan).

## *Periodic-Acid Schiff (PAS) staining and analysis*

For PAS staining, staining kit (G1281, Solarbio, Beijing, China) is used. About 1% periodic acid is oxidized for 10 min, and rinsed 3 times with distilled water, 5 min each. Reducing solution is added for 1 min and then Schiff's solution for 25 min. Washing is done with tap water for 5-10 min, and xylenin is added for 1 min, following rinsing with tap water. Thereafter, dehydration and clearing are done using an alcohol gradient and xylene as described above. Mounting and photos are also carried out as described above.

For analysis of sections at 200X magnification, 15-20 glomeruli were randomly selected from each kidney. For each glomerulus, the mesangial area was determined by the quantification of the PAS-positive and nucleus-free areas in the mesangial region, and Image-Pro Plus 6.0 software (Image-pro Plus, Media Cybernetics, Inc., USA) was used to conduct statistical analysis on the proportion of mesangial area of each glomerulus to the total area[[1](#_ENREF_1), [2](#_ENREF_2)].

## *Masson's Trichrome staining and Analysis*

Masson’s Trichrome Stain Kit (G1240, Solarbio, Beijing, China) is applied. The processed paraffin sections were stained with hematoxylin dye for 5min and washed with running water for 10min. Following this 1% hydrochloric acid ethanol was added to for 5s and slides were again rinsed with distilled water for 5min. Acid fuchsin solution of lichun red was added for 15min. A wash was then done with a weak acid working solution for 6s. Phosphomolybdate aqueous solution was added for 2min. The slides were washed with a weak acid working solution for 6s and an aniline blue solution was added for 40s. Finally, the slides were exposed to a weak acid solution for 1min, and rinsed with distilled water twice. Treated slides were dehydrated, cleared and mounted as described above.

For quantification of interstitial fibrosis using Masson’s trichrome stain, 6-10 randomly-selected cortical fields from each animal under a 200X magnification were analyzed using Image-Pro Plus 6.0 software. The proportion of blue-stained area was quantified as described previously[[3](#_ENREF_3)].

[1] R. Nishad, et al., Growth hormone induces Notch1 signaling in podocytes and contributes to proteinuria in diabetic nephropathy, J Biol Chem 294(44) (2019) 16109-16122.

[2] T.W. Tervaert, et al., Pathologic classification of diabetic nephropathy, J Am Soc Nephrol 21(4) (2010) 556-63.

[3] W. Wang, et al., Deletion of scavenger receptor A protects mice from progressive nephropathy independent of lipid control during diet-induced hyperlipidemia, Kidney Int 81(10) (2012) 1002-1014.
