# Supplementary material for: Amelioration of diabetic nephropathy in mice by a single intravenous injection of human mesenchymal stromal cells at early and later disease stages is associated with restoration of autophagy
Source: Stem Cell Res Ther. 2024 Mar 5;15:66. doi: 10.1186/s13287-024-03647-x (PMC10916232; doi:10.1186/s13287-024-03647-x)
Supplement: Supplementary file 3 — Additional file 3. Figure S1. Full-length gels and blots of Figure 8C. [file 13287_2024_3647_MOESM3_ESM.pdf]

Full-length blots

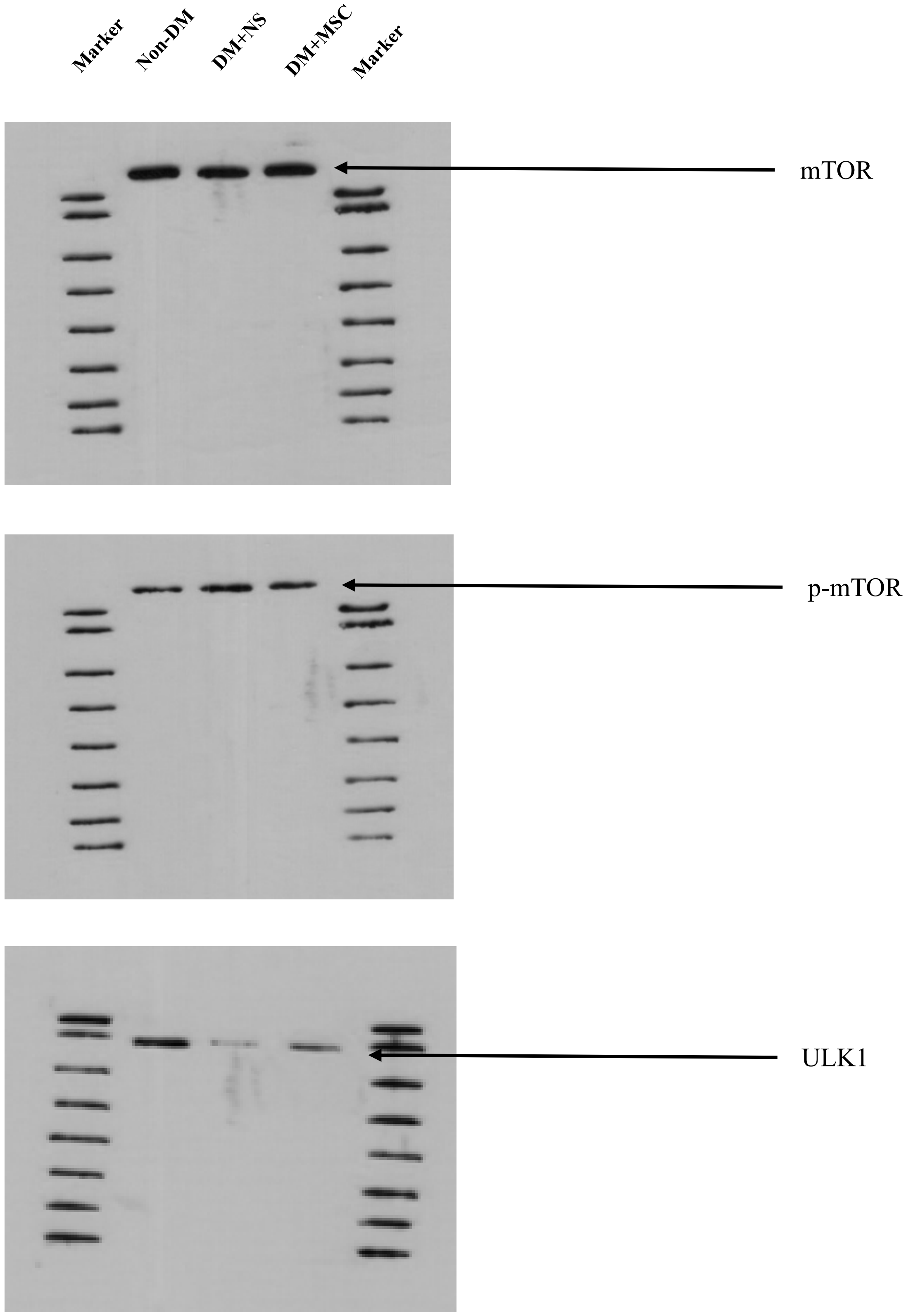

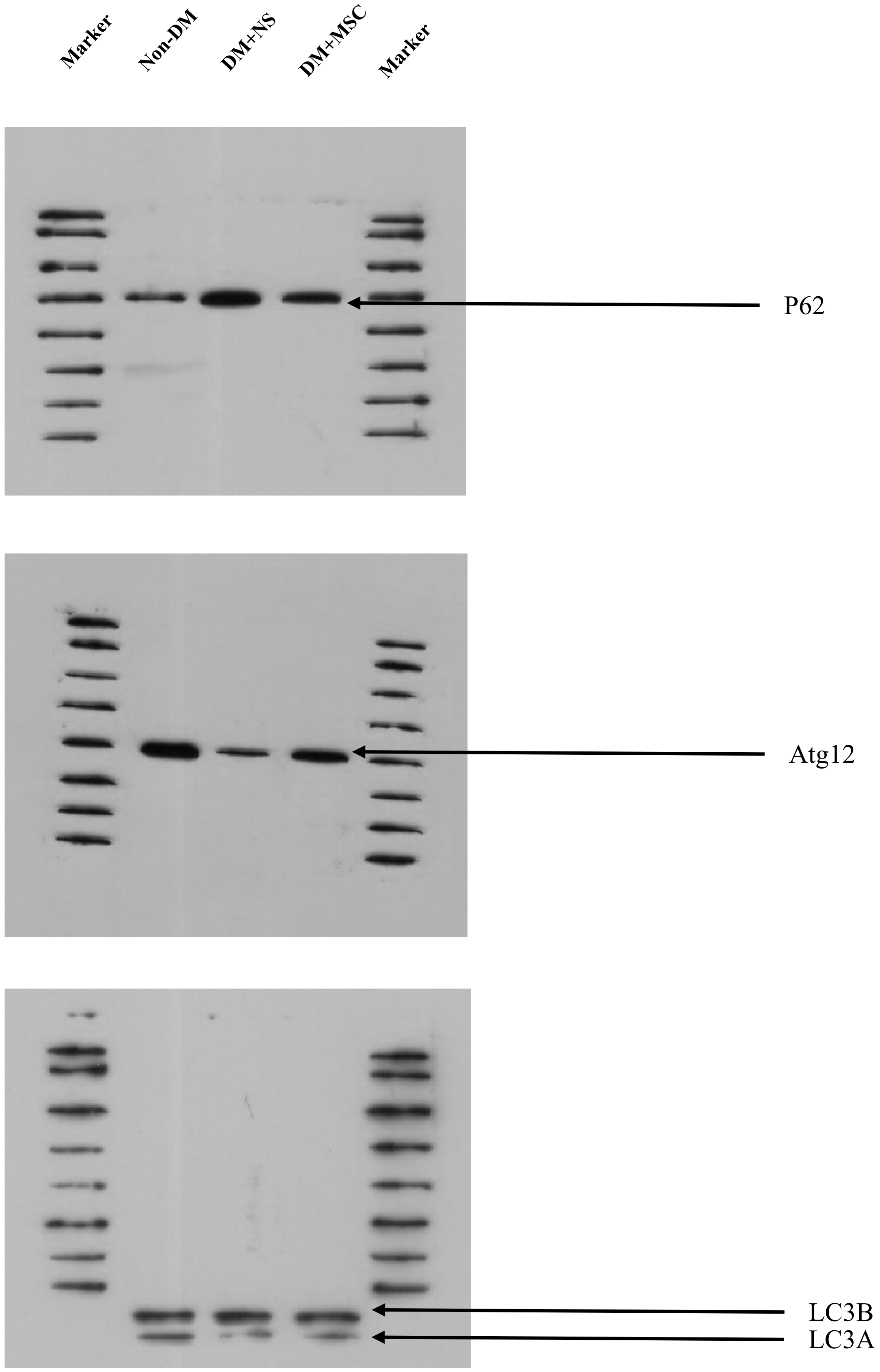
Marker
Non-DM
DM+NS
DM+MSC
Marker
P62
Atg12
LC3B
LC3A

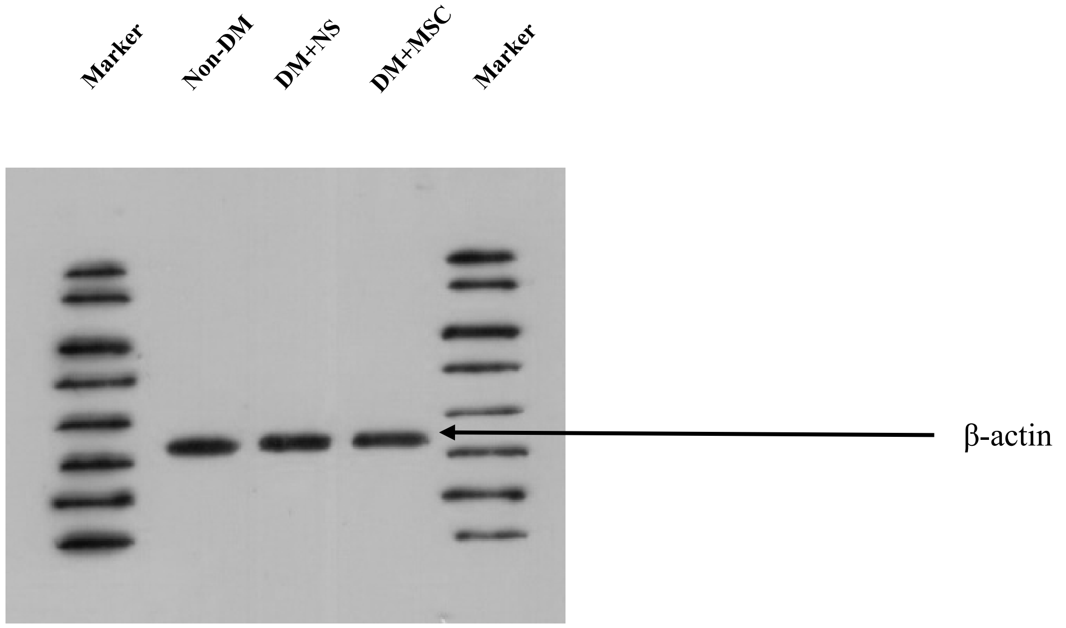

Supplementary Figure S1. **Full-length blots of Figure 8C (10 weeks).** For detecting mTOR, p-mTOR, ULK1, Atg12, p62, and LC3A/B, the PVDF membrane were probed with antibodies against mTOR, p-mTOR, ULK1, Atg12, p62, LC3A/B, GAPDH, and β-actin, respectively. For each target protein, the probed-membrane was stripped by stripping buffer (Cat. No. 46430, Thermo Fisher Scientific) after detected by the β-actin antibody, and then re-probed with the target antibody again. The blots of target protein were indicated with an arrow.

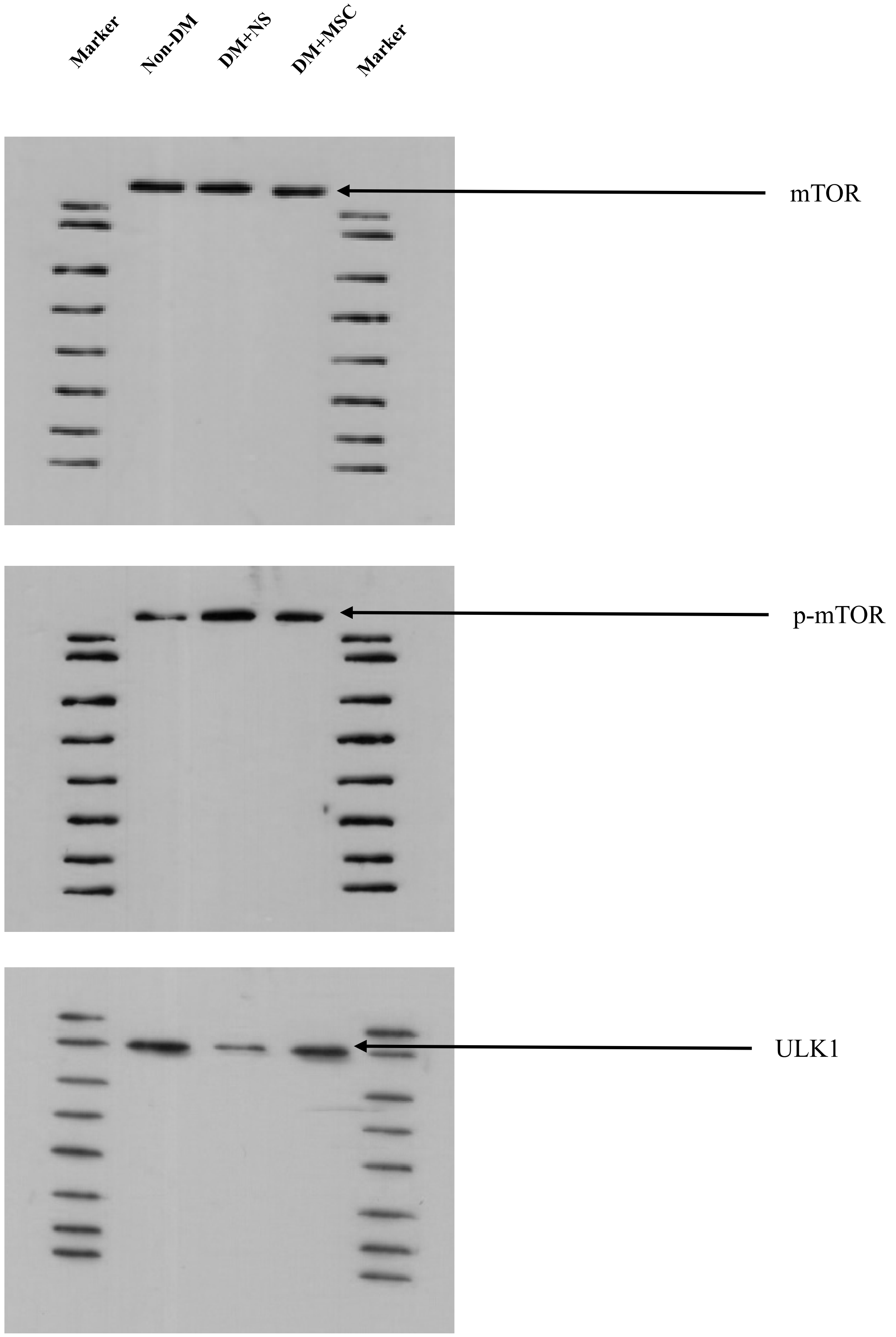
Marker
Non-DM
DM+NS
DM+MSC
Marker
mTOR
p-mTOR
ULK1

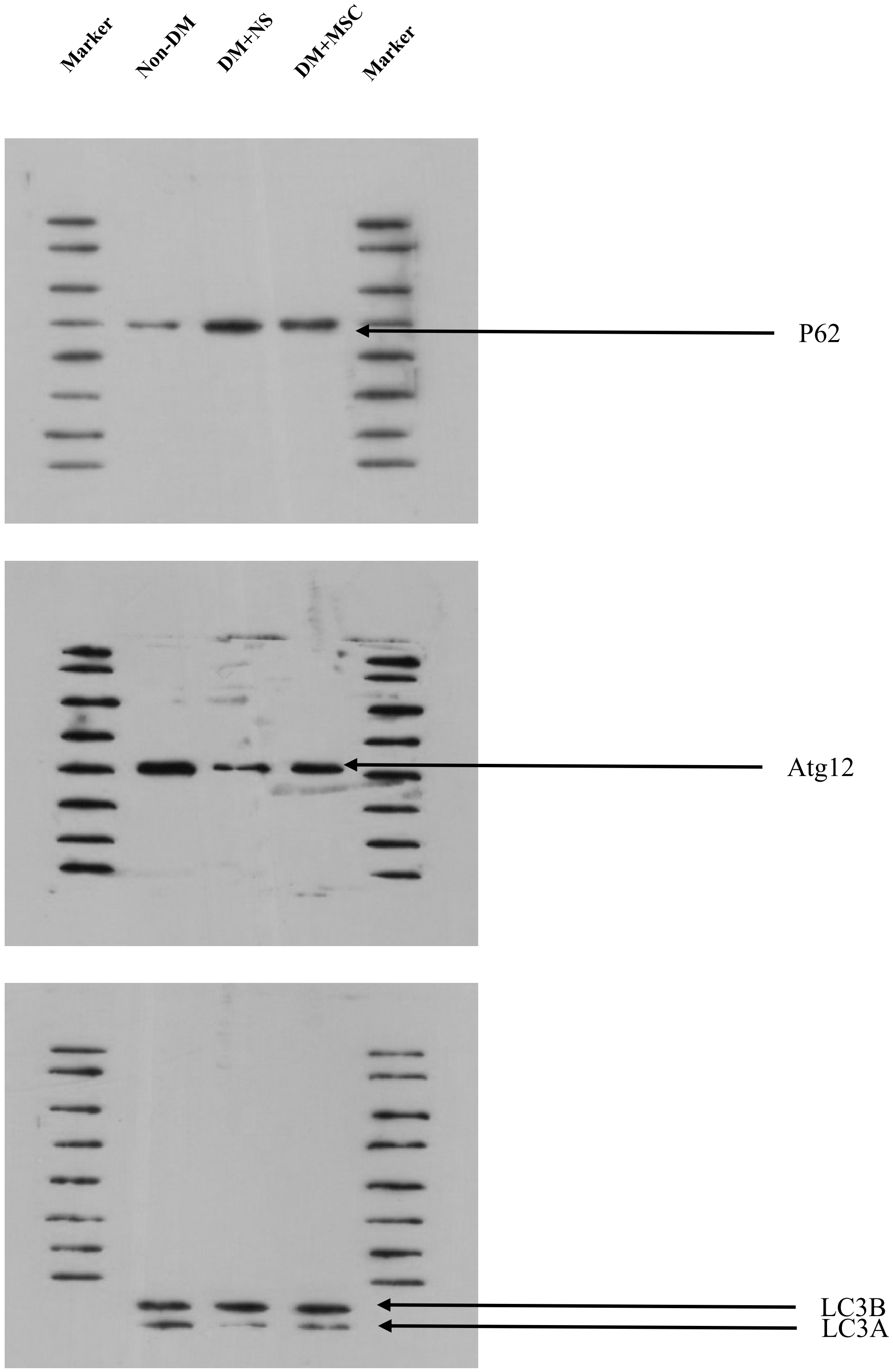

Supplementary Figure S1. **Full-length blots of Figure 8C (18 weeks).** For detecting mTOR, p-mTOR, ULK1, Atg12, p62, and LC3A/B, the PVDF membrane were probed with antibodies against mTOR, p-mTOR, ULK1, Atg12, p62, LC3A/B, GAPDH, and β-actin,

respectively. For each target protein, the probed-membrane was stripped by stripping buffer (Cat. No. 46430, Thermo Fisher Scientific) after detected by the β-actin antibody, and then re-probed with the target antibody again. The blots of target protein were indicated with an arrow.
